# Syntheses and crystal structures of a new pyrazine dicarboxamide ligand, *N* ^2^,*N* ^3^-bis­(quinolin-8-yl)pyrazine-2,3-dicarboxamide, and of a copper perchlorate binuclear complex

**DOI:** 10.1107/S2056989020001838

**Published:** 2020-02-14

**Authors:** Dilovan S. Cati, Helen Stoeckli-Evans

**Affiliations:** a Debiopharm International S.A., Chemin Messidor 5-7, CH-1002 Lausanne, Switzerland; b Institute of Physics, University of Neuchâtel, rue Emile-Argand 11, CH-2000 Neuchâtel, Switzerland

**Keywords:** crystal structure, pyrazine, dicarboxamide, quinoline, copper(II), bis-tridentate coordination, hydrogen bonding, offset π–π inter­actions, Hirshfeld surface analysis

## Abstract

The title pyrazine dicarboxamide ligand, *N* ^2^,*N* ^3^-bis­(quinolin-8-yl)pyrazine-2,3-dicarboxamide (**H_2_L1**), C_24_H_16_N_6_O_2_, has a twisted conformation with the outer quinoline groups being inclined to the central pyrazine ring by 9.00 (6) and 78.67 (5)°, and by 79.94 (4)° to each other. In the crystal, molecules are linked by C—H⋯O hydrogen bonds, forming layers parallel to the (10) plane, which are in turn linked by offset π–π inter­actions [inter­centroid distances 3.4779 (9) and 3.6526 (8) Å], forming a supra­molecular three-dimensional structure. Reaction of the ligand **H_2_L1** with Cu(ClO_4_)_2_ in aceto­nitrile leads to the formation of the binuclear complex, [μ-(3-{hy­droxy[(quinolin-8-yl)imino]­meth­yl}pyrazin-2-yl)[(quinolin-8-yl)imino]­methano­lato]bis­[diaceto­nitrile­copper(II)] tris­(per­chlor­ate) aceto­nitrile disolvate, [Cu_2_(C_24_H_15_N_6_O_2_)(CH_3_CN)_4_](ClO_4_)_3_·2CH_3_CN or [Cu_2_(HL1^−^)(CH_3_CN)_4_](ClO_4_)_3_·2CH_3_CN (**I**). In the cation of complex **I**, the ligand coordinates to the copper(II) atoms in a bis-tridentate fashion. A resonance-assisted O—H⋯O hydrogen bond is present in the ligand; the position of this H atom was located in a difference-Fourier map. Both copper(II) atoms are fivefold coordinate, being ligated by three N atoms of the ligand and by the N atoms of two aceto­nitrile mol­ecules. The first copper atom has a perfect square-pyramidal geometry while the second copper atom has a distorted shape. In the crystal, the cation and perchlorate anions are linked by a number of C—H⋯O hydrogen bonds, forming a supra­molecular three-dimensional structure.

## Abstract 2

On reaction of the new pyrazine dicarboxamide ligand, *N* ^2^,*N* ^3^-bis­(quinolin-8-yl)pyrazine-2,3-dicarboxamide, with Cu(ClO_4_)_2_ in aceto­nitrile a binuclear copper complex was formed. Both copper atoms are CuN_5_ coordinate, and while one copper atom has a perfect square-pyramidal coordination sphere the other has a distorted shape.

## Chemical context   

The title ligand, *N* ^2^,*N* ^3^-bis­(quinolin-8-yl)pyrazine-2,3-dicarboxamide (**H_2_L1**), is very similar to ligand *N^2^*-[(2,3-di­hydro­pyridin-2-yl)meth­yl]-*N^3^*-(pyridin-2-ylmeth­yl)pyrazine-2,3-di­carboxamide (**H_2_L2**), for which two polymorphs have been reported (Cati *et al.*, 2004 ▸; Cati & Stoeckli-Evans, 2004 ▸). These and other pyrazine-carboxamide ligands were synthesized to explore their coordination behaviour with first-row transition metals and to study the magnetic exchange behaviour of the complexes (Cati, 2002 ▸). With ligand **H_2_L2**, grid [2 × 2] complexes have been synthesized using Cu(BF_4_)_2_ (Hausmann *et al.*, 2003 ▸), and with Cu(ClO_4_)_2_ and NiCl_2_ (Cati *et al.*, 2004 ▸). The latter complexes were shown to exhibit multiple anion encapsulation and anti­ferromagnetic exchange behaviour. In all of these complexes, the ligand is monodeprotonated and the bis-tridentate coordinated ligands have relatively planar conformations. Herein, we report on the syntheses and crystal structures of the title pyrazine dicarboxamide ligand (**H_2_L1**), and of a binuclear copper complex, **I**, which was synthesized by the reaction of **H_2_L1** with copper perchlorate using aceto­nitrile as solvent. The various inter­molecular contacts in the crystal of **H_2_L1** have been studied by Hirshfeld surface analysis.
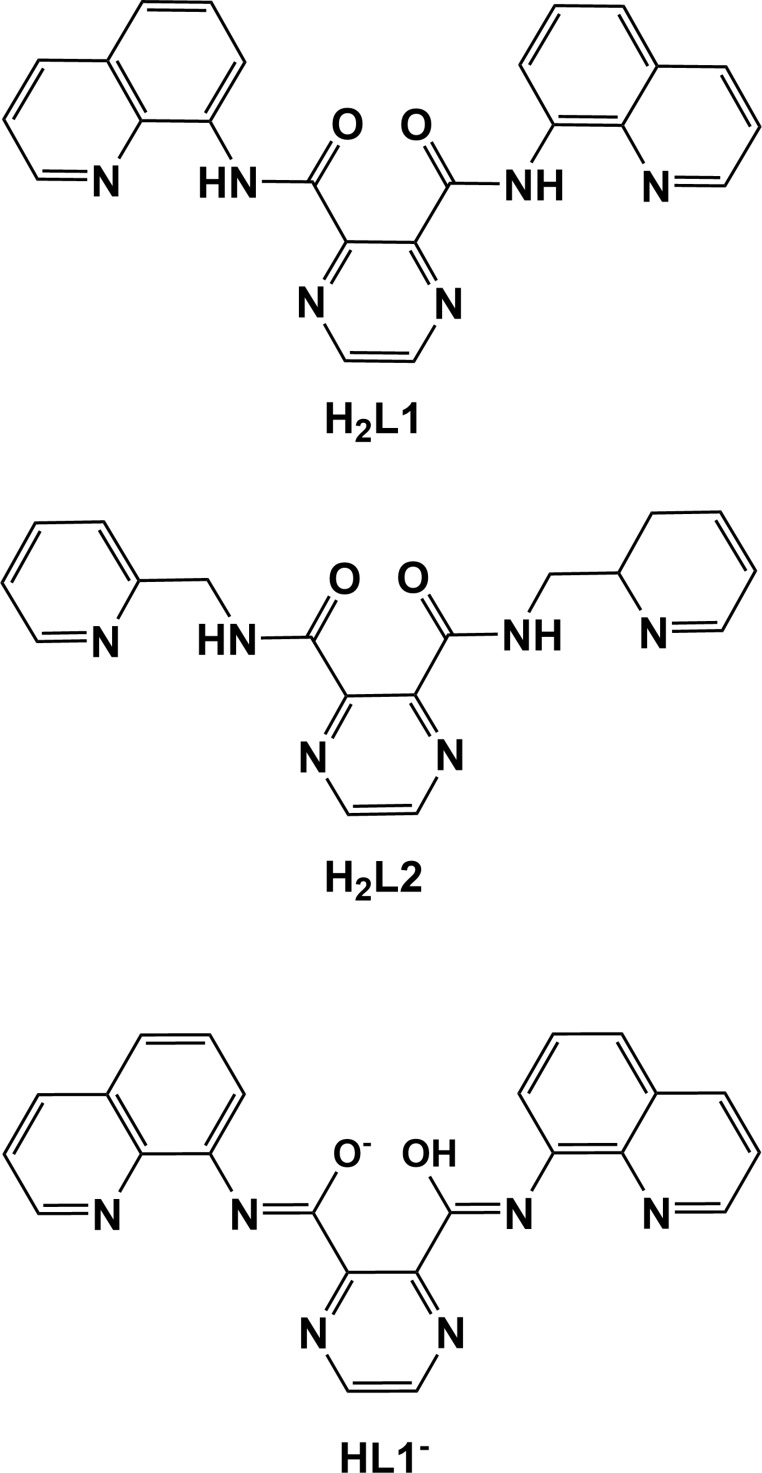
 
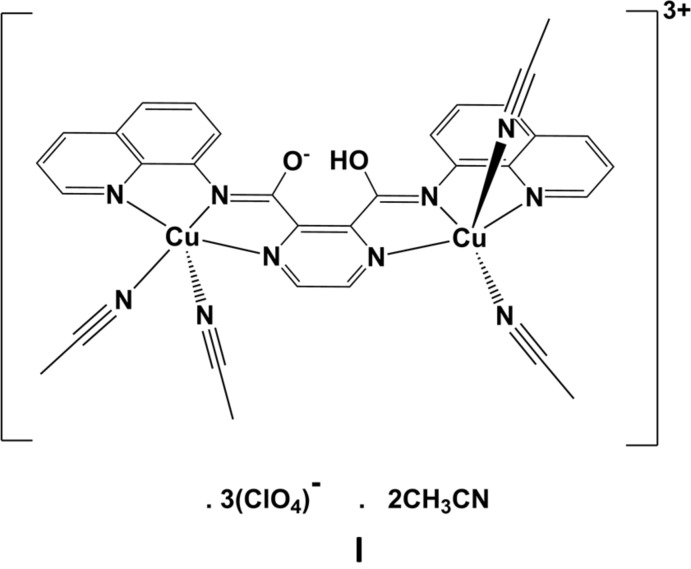


## Structural commentary   

**Figure 1:**
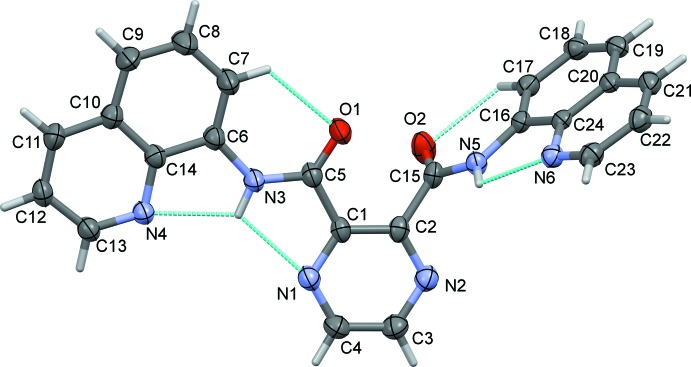
Mol­ecular structure of ligand **H_2_L1**, with atom labelling. Displacement ellipsoids are drawn at the 50% probability level. The intra­molecular contacts are shown as dashed lines (see Table 1 ▸).

The mol­ecular structure of ligand **H_2_L1** is illustrated in Fig. 1 ▸. The quinoline ring (N4/C6–C14, r.m.s. deviation 0.008 Å) is inclined to the pyrazine ring (N1/N2/C1–C4) by 9.00 (6)°. The NH hydrogen atom H3*N* is involved in two intra­molecular N—H⋯N contacts (Fig. 1 ▸, Table 1 ▸). On the opposite side of the mol­ecule, the quinoline ring system (N6/C16–C124, r.m.s. deviation 0.009 Å) is inclined to the pyrazine ring by 78.67 (5)°, with a single intra­molecular N—H⋯N contact (Fig. 1 ▸, Table 1 ▸). Both carboxamide O atoms, O1 and O2, are involved in short C—H⋯O intra­molecular contacts, enclosing *S*(6) ring motifs (Fig. 1 ▸, Table 1 ▸). Hence, the mol­ecule is L-shaped with the two quinoline ring systems being inclined to each other by 79.94 (4)°.

**Figure 2:**
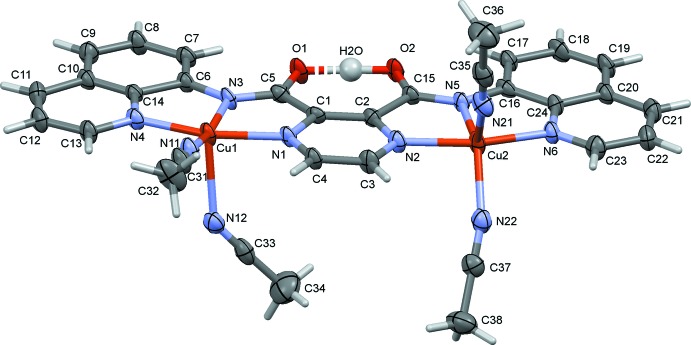
Mol­ecular structure of the cation of complex **I**, with atom labelling. Displacement ellipsoids are drawn at the 50% probability level. For clarity, the perchlorate anions and the solvate aceto­nitrile mol­ecules have been omitted.

**Figure 3:**
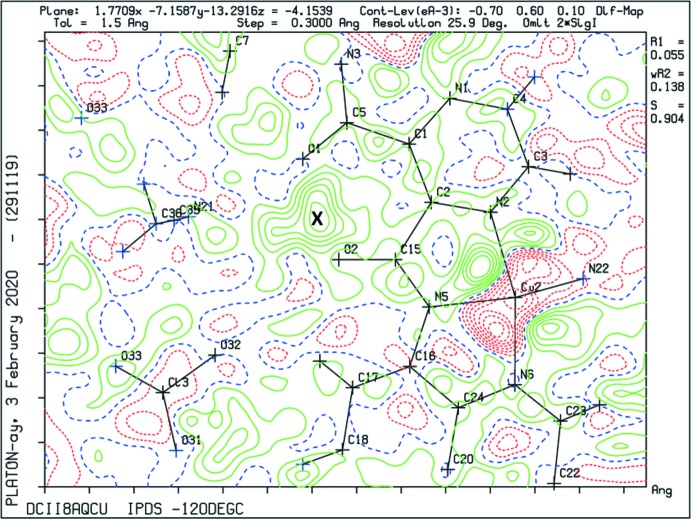
A difference-Fourier map showing the position (*X*) of the hydroxyl H atom, H2*O*, in complex **I**.

In the binuclear copper complex **I** (Fig. 2 ▸), which was formed by the reaction of **H_2_L1** with Cu(ClO_4_)_2_·2H_2_O, the bond lengths and angles involving the two amide moieties (Table 2 ▸; notably the bond lengths involving atoms C5 and C15) indicate that the situation in the crystal resembles that shown in the scheme for **HL1^−^**. On coordinating to two metal ions the ligand **H_2_L1** becomes negatively charged, and is stabilized by a hydrogen bond to the adjacent neutral amide tautomer. In order to locate the H atom of this resonance-assisted O—H⋯O hydrogen bond (Table 4 ▸), and as recommended by Fábry (Fábry, 2018 ▸) and Spek (Spek, 2020 ▸), a difference-Fourier map (Fig. 3 ▸) was examined and the position of the H atom was located closest to atom O2.

The asymmetric unit of compound **I** is composed of the binuclear 3^+^ cation, three perchlorate anions and two aceto­nitrile solvate mol­ecules. In the cation (Fig. 2 ▸), the ligand coordinates to the copper(II) atoms in a bis-tridentate fashion. Selected bond lengths and angles involving atoms Cu1 and Cu2 are given in Table 3 ▸. Atom Cu1 has a perfect square-pyramidal fivefold CuN_5_ coordination sphere with a τ_5_ value of 0.0 (τ_5_ = 0 for an ideal square-pyramidal coordination sphere, and = 1 for an ideal trigonal–pyramidal coordination sphere; Addison *et al.*, 1984 ▸). The Cu—N bond lengths in the equatorial plane vary from 1.936 (3) to 2.013 (4) Å, while the apical Cu—N12 bond length is 2.266 (4) Å. Atom Cu2 also has a fivefold CuN_5_ coordination sphere but the value of τ_5_ is 0.38, indicating a distorted shape. The Cu—N bond lengths in the approximate equatorial plane vary from 1.943 (4) to 2.054 (4) Å, while the apical Cu—N21 bond length is 2.137 (4) Å. The ligand is essentially planar with the quinoline ring systems (involving atoms N4 and N6) being inclined to the central pyrazine ring by 1.78 (17) and 1.80 (17)°, respectively, and by 2.65 (13)° to each other.

## Supra­molecular features   

**Figure 4:**
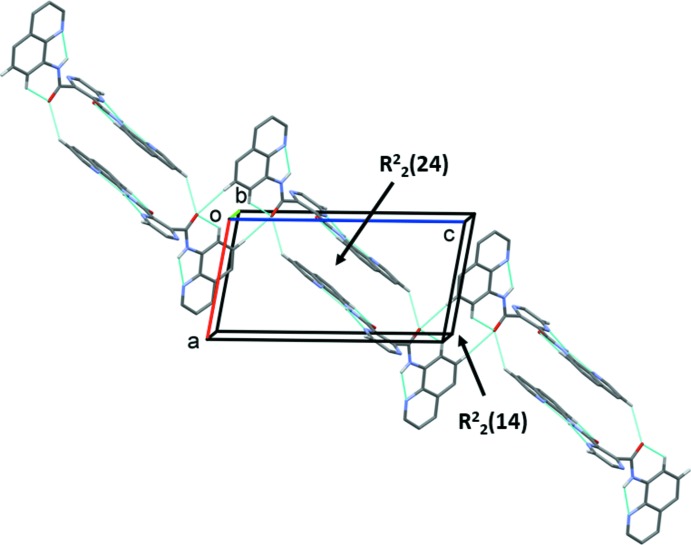
A partial view along the *b* axis of the crystal packing of ligand **H_2_L1**. Hydrogen bonds are shown as dashed lines (see Table 1 ▸). For clarity, in this and subsequent crystal packing figures, only the H atoms involved in these inter­actions have been included.

**Figure 5:**
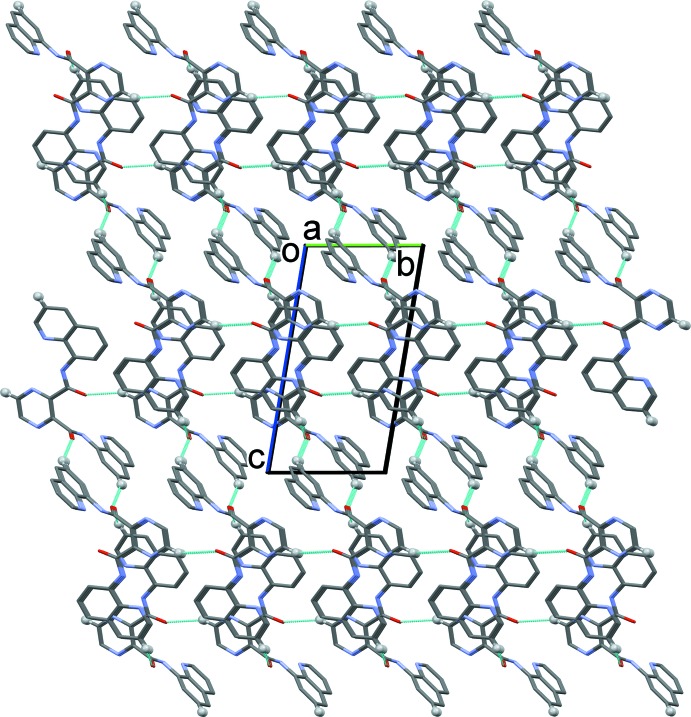
The crystal packing of ligand **H_2_L1**, viewed along the *a* axis. Hydrogen bonds are shown as dashed lines (see Table 1 ▸).

In the crystal of **H_2_L1**, mol­ecules are linked by two pairs of C—H⋯O hydrogen bonds (C12—H12⋯O2^ii^ and C18—H18⋯O2^iii^), each involving inversion-related mol­ecules, forming chains of loops propagating along the [10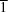] direction. The loops enclose 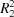
(14) and 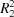
(24) ring motifs (see Fig. 4 ▸, Table 1 ▸). A third C—H⋯O hydrogen bond (C4—H4⋯O1^i^) links the chains in the *b*-axis direction (Table 1 ▸), so forming layers lying parallel to the (10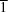) plane. Finally the layers are linked by offset π–π inter­actions involving the pyrazine ring and an inversion-related quinoline ring system, and by inversion-related quinoline ring systems, so forming a supra­molecular three-dimensional structure (Fig. 5 ▸). The first offset π–π inter­action involves pyrazine ring N1/N2/C1–C4 (centroid *Cg*1) and quinoline ring system N4/C6–C14 (centroid *Cg*2) with *Cg*1⋯*Cg*2^i^ = 3.4779 (9) Å, α = 9.00 (6)°, β = 17.5 °, γ = 11.3°; the inter­planar distances are 3.4106 (6) and 3.3162 (5) Å, with an offset of 1.048 Å [symmetry code: (i) −*x*, −*y* + 2, −*z* + 1]. The second offset π–π inter­action involves inversion-related N6/C16–C24 (centroid *Cg*3) quinoline ring systems with *Cg*3⋯*Cg*3^ii^ = 3.6526 (8) Å, α = 0.00 (4)°, β = 24.1°, γ = 24.1°, inter­planar distance = 3.3333 (4) Å, with an offset of 1.494 Å [symmetry code: (ii) −*x* − 1, −*y* + 1, −*z*].

**Figure 6:**
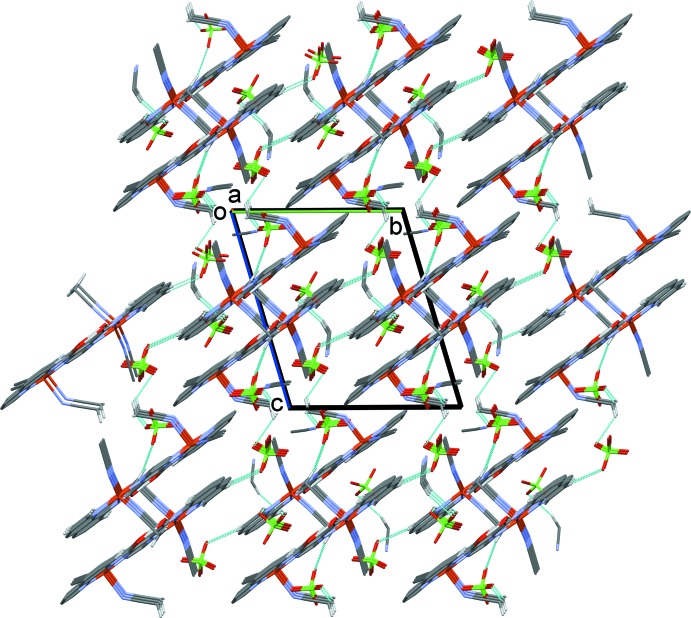
The crystal packing of complex **I**, viewed along the *a* axis. Hydrogen bonds are shown as dashed lines (see Table 4 ▸).

In the crystal of complex **I**, the cations are arranged in layers parallel to the (012) plane. They are linked *via* the perchlorate anions by a number of C—H⋯O hydrogen bonds (Table 4 ▸), so forming a supra­molecular three-dimensional structure (Figs. 6 ▸ and 7 ▸). There is only one significant C—H⋯N hydrogen bond present involving the solvate aceto­nitrile N atom, N31, linking it to the CH_3_ group of a coordinated aceto­nitrile mol­ecule on atom Cu2 (Table 4 ▸).

## Hirshfeld surface analysis of ligand HL1   

**Figure 8:**
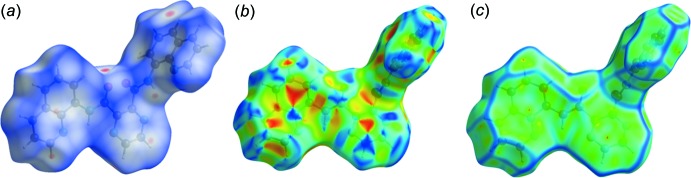
(*a*) A view of the Hirshfeld surface for **H_2_L1** mapped over *d*_norm_, with colour code −0.1884 to 1.1906 a.u., (*b*) a view of the Hirshfeld surface mapped over the shape-index for **H_2_L1**, (*c*) a view of the Hirshfeld surface mapped over the curvedness for **H_2_L1**.

The Hirshfeld surface analysis (Spackman & Jayatilaka, 2009 ▸) and the associated two-dimensional fingerprint plots (McKinnon *et al.*, 2007 ▸) were performed with *CrystalExplorer17* (Turner *et al.*, 2017 ▸). A view of the Hirshfeld surface of **H_2_L1** mapped over *d*_norm_ is shown in Fig. 8 ▸ *a*, where short inter­atomic contacts are indicated by the faint red spots. The π–π stacking in the crystal is confirmed by the small blue regions surrounding bright-red spots in the various aromatic rings in Fig. 8 ▸ *b*, the Hirshfeld surface mapped over the shape-index. The π–π stacking is also confirmed by the flat regions around the aromatic units in Fig. 8 ▸ *c*, the Hirshfeld surface mapped over the curvedness.

**Figure 9:**
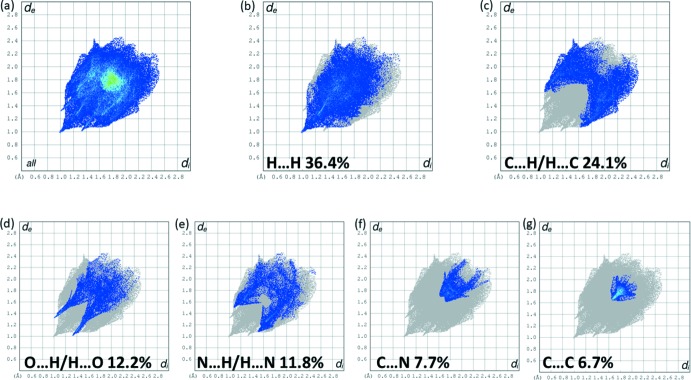
(*a*) The full two-dimensional fingerprint plot for **H_2_L1** and the fingerprint plots delineated into (*b*) H⋯H, (*c*) C⋯H/H⋯C, (*d*) O⋯H/H⋯O, (*e*) N⋯H/H⋯N, (*f*) C⋯N, (*g*) C⋯C contacts.

The two-dimensional fingerprint plots for **H_2_L1** are given in Fig. 9 ▸. The principal inter­molecular contact types are delineated into H⋯H at 36.4% (Fig. 9 ▸ *b*), C⋯H/H⋯C at 24.1% (Fig. 9 ▸ *c*), O⋯H/H⋯O at 12.2% (Fig. 9 ▸ *d*) and N⋯H/H⋯N at 11.8% (Fig. 9 ▸ *e*) contacts. The contributions of the C⋯N (Fig. 9 ▸ *f*) and C⋯C (Fig. 9 ▸ *g*) contacts are 7.7 and 6.7%, respectively.

## Database survey   

A search of the Cambridge Structural Database (CSD, Version 5.41, update November 2019; Groom *et al.*, 2016 ▸) for pyrazine carboxamides including a quinoline group yielded 28 hits. Many of these structures concern the ligand *N*-(quinolin-8-yl)pyrazine-2-carboxamide (CSD refcode EFODIP; Cati & Stoeckli-Evans, 2019 ▸) and metal complexes of this ligand, such as (acetato)[*N*-(quinolin-8-yl)pyrazine-2-carboxamidato]copper(II) monohydrate (AYIFOF; Meghdadi *et al.*, 2013 ▸) and hexa­kis­(μ-acetato)­bis­(methanol)bis­[*N*-(quniolin-8-yl)pyrazine-2-carboxamide]­tetra­copper(II) methanol solvate (EFODOV; Cati & Stoeckli-Evans, 2019 ▸). However, the majority of the structures are hetero bimetallic iron–mangan­ese cyano complexes that exhibit super-exchange magnetic properties (see file S1 in the supporting information).

## Synthesis and crystallization   

**Synthesis of the ligand** ***N^2^,N^3^*** **-di(quinolin-8-yl)pyrazine-2,3-dicarboxamide (H_2_L1):** 8-amino­quinoline (3.18g, 22 mmol) was added to a solution of pyrazine-2,3-di­carb­oxy­lic acid (1.68g, 10 mmol) and 1,1′-carbonyl­diimidazole (4.20g, 26 mmol) in 180 ml of DMF (anhydride) in a two-necked flask (500 ml). The solution was mixed for 15 min at room temperature and then heated gradually for 1 h and then refluxed for 7 h. The reaction mixture was then cooled and added directly to a column (10 g of SiO_2_, diameter of the column 1 cm), and eluted with DMF. After evaporation of the solvent the solid obtained was refluxed in 80 ml of ethanol for 10 min and then filtered. The brown–yellow solid obtained was recrystallized from DMF and on slow evaporation of the solvent pale-yellow rod-like crystals of **H_2_L1** were obtained (yield 22%; m.p. 569 K). ^1^H NMR (400 MHz, DMSO-*d*_6_): 11.47 (*s*, 1H, HN_3_); 9.04 (*s*, 1H, H3 = H4); 8.95 (*dd*, 1H, *J*_13,12_ = 4.2, *J*_13,11_ = 1.7, H13); 8.82 (*dd*, 1H, *J*_7,8_ = 7.7, *J*_7,9_ = 1.2, H7); 8.47 (*dd*, 1H, *J*_11,12_ = 8.3, *J*_11,13_ = 1.7, H11); 7.77 (*dd*, 1H, *J*_9,8_ = 8.3, *J*_9,7_ = 1.2, H9); 7.66 (*m*, 2H, H12 & H8). IR (KBr pellet, cm^−1^): 3350 (*s*), 3300 (*s*), 1678 (*vs*), 1560 (*vs*), 1530 (*vs*), 1520 (*vs*), 1488 (*vs*), 1465 (*s*), 1427 (*vs*), 1385 (*s*), 1326 (*s*), 1151 (*s*), 1109 (*s*), 919 (*s*), 829 (*vs*), 792 (*vs*), 752 (*s*), 652 (*s*), 607 (*s*). Analysis. for C_24_H_16_N_6_O_2_ (Mr = 420.43 g mol^−1^) calculated (%) C: 68.56, H: 3.84, N: 19.99; found (%) C: 68.70, H: 3.92, N: 20.40.

**Synthesis of complex [Cu_2_(HL^−^)(CH_3_CN)_4_]**·**3(ClO_4_)**·**2(CH_3_CN) (I):** Cu(ClO_4_)_2_·6H_2_O (28 mg, 0.075 mmol) and **H_2_L** (15 mg, 0.036 mmol) were added to 10 ml of aceto­nitrile. The green solution obtained was stirred at room temperature for 10 min, then left at ambient temperature. After slow evaporation of the solvent green plate-like crystals of **I** were obtained (yield: 15 mg, 40%). IR (KBr pellet, cm^−1^): 1660 (*vs*), 1645 (*vs*), 1615 (*vs*), 1581 (*s*), 1566 (*s*), 1389 (*s*), 1147 (*s*), 1089 (*vs*), 625 (*s*).

**Figure 10:**
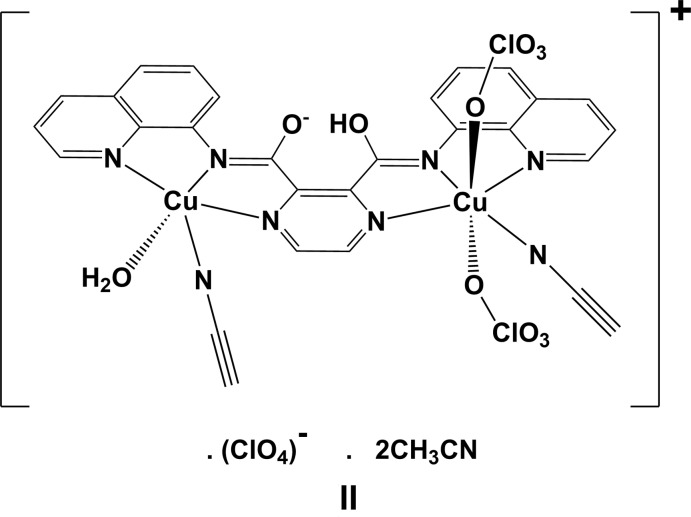
The structure of complex [(H_2_O)Cu_2_(HL1^−^)(ClO_4_)_2_(CH_3_CN)]·(ClO_4_)·2(CH_3_CN) (**II**).

During this experiment, two types of crystals were obtained on slow evaporation of the filtrate of the reaction mixture; green plate-like crystals of the binuclear complex **I** and thin colourless crystals of a second binuclear complex, **[(H_2_O)Cu_2_(HL1^−^)(ClO_4_)_2_(CH_3_CN)]**·**(ClO_4_)**·2**(CH_3_CN)** (**II**). The data set for **II**, measured at 153 K, has only 20% observed data; the crystal did not diffract beyond 20° in θ. While the structure is perfectly clear (Fig. 10 ▸), the analysis is probably at the limit of being acceptable: *R*_int_ = 0.36 and *GoF* = 0.43, with the s.u.s. of the Cu—O/N bond lengths varying between 0.008 and 0.014 Å. The final values of *R*[*F* ^2^ > 2σ(*F* ^2^)] and *wR*(*F* ^2^) are 0.0558 and 0.1328. The CIF, including the HKL file, has been deposited with the Cambridge Structural Database (refcode XUFZAC; CCDC 1981495; Groom *et al.*, 2016 ▸). It is supplied here as supporting information file S2.

## Refinement   

Crystal data, data collection and structure refinement details are summarized in Table 5 ▸. For ligand **H_2_L1**, the NH H atoms were located in a difference-Fourier map and freely refined. For both **H_2_L1** and complex **I**, the C-bound H atoms were included in calculated positions and refined as riding: C—H = 0.94–0.98 Å with *U*_iso_(H) = 1.5*U*_eq_(C-meth­yl) and 1.2*U*_eq_(C) for other H atoms. For complex **I**, a resonance assisted O2—H2*O*⋯O1 hydrogen bond (Table 4 ▸) is present in the ligand; the position of the H atom, H2*O*, was located closest to atom O2 in a difference-Fourier map (Fig. 3 ▸) and was freely refined.

With the STOE IPDS I, a one-circle diffractometer, for the triclinic system often only 93% of the Ewald sphere is accessible. Hence, for complex **I** the ‘diffrn_reflns_Laue_measured_fraction_full’ of 0.941 is below the required minimum of 0.95.

## Supplementary Material

Crystal structure: contains datablock(s) H2L1, I, Global. DOI: 10.1107/S2056989020001838/zl2771sup1.cif

Structure factors: contains datablock(s) H2L1. DOI: 10.1107/S2056989020001838/zl2771H2L1sup2.hkl

Structure factors: contains datablock(s) I. DOI: 10.1107/S2056989020001838/zl2771Isup3.hkl

CSD search. DOI: 10.1107/S2056989020001838/zl2771sup4.pdf

Crystal structure: contains datablock(s) II. DOI: 10.1107/S2056989020001838/zl2771sup5.cif

CCDC references: 1982863, 1982862

Additional supporting information:  crystallographic information; 3D view; checkCIF report

## Figures and Tables

**Figure 7:**
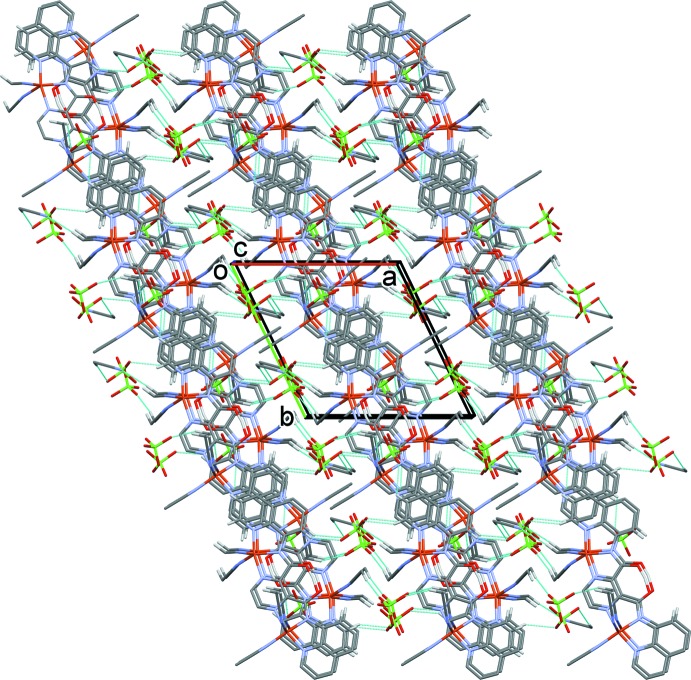
The crystal packing of complex **I**, viewed along the *c* axis. Hydrogen bonds are shown as dashed lines (see Table 4 ▸).

**Table 1:** Hydrogen-bond geometry (Å, °) for **H_2_L1** [Chem scheme1]

*D*—H⋯*A*	*D*—H	H⋯*A*	*D*⋯*A*	*D*—H⋯*A*
N3—H3*N*⋯N1	0.88 (2)	2.256 (16)	2.6791 (18)	109 (1)
N3—H3*N*⋯N4	0.88 (2)	2.218 (16)	2.6657 (16)	111 (1)
N5—H5*N*⋯N6	0.85 (2)	2.233 (16)	2.6759 (17)	113 (1)
C7—H7⋯O1	0.94	2.31	2.9136 (19)	122
C17—H17⋯O2	0.94	2.28	2.8818 (18)	122
C4—H4⋯O1^i^	0.94	2.57	3.4249 (18)	151
C12—H12⋯O2^ii^	0.94	2.60	3.3589 (19)	138
C18—H18⋯O2^iii^	0.94	2.48	3.3289 (19)	151

Symmetry codes: (i) 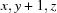; (ii) 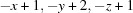; (iii) 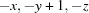.

**Table 2:** A comparison of bond lengths and angles (Å, °) in the carboxamide units of **H_2_L1** and **I**

	**H_2_L1**	**I**
O1—C5	1.222 (2)	1.289 (5)
N3—C5	1.347 (2)	1.319 (6)
C1—C5	1.506 (2)	1.477 (7)
O2—C15	1.219 (2)	1.267 (5)
N5—C15	1.348 (2)	1.310 (6)
C2—C15	1.508 (2)	1.518 (5)
		
N3—C5—O1	125.78 (13)	123.9 (4)
N3—C5—C1	113.91 (12)	114.4 (4)
O1—C5—C1	120.32 (12)	121.7 (4)
N5—C15—O2	125.13 (13)	125.7 (4)
N5—C15—C2	115.76 (12)	112.1 (4)
O2—C15—C2	118.87 (12)	122.1 (4)

**Table 3:** Selected geometric parameters (Å, °) for **I** [Chem scheme1]

Cu1—N1	2.013 (4)	Cu2—N2	1.996 (4)
Cu1—N3	1.936 (3)	Cu2—N5	1.943 (4)
Cu1—N4	1.952 (4)	Cu2—N6	1.961 (3)
Cu1—N11	1.982 (4)	Cu2—N21	2.137 (4)
Cu1—N12	2.266 (4)	Cu2—N22	2.054 (4)
			
N4—Cu1—N1	163.76 (14)	N6—Cu2—N2	163.51 (16)
N3—Cu1—N11	163.66 (16)	N5—Cu2—N22	140.74 (16)

**Table 4:** Hydrogen-bond geometry (Å, °) for **I** [Chem scheme1]

*D*—H⋯*A*	*D*—H	H⋯*A*	*D*⋯*A*	*D*—H⋯*A*
O2—H2*O*⋯O1	1.12 (6)	1.29 (6)	2.397 (4)	169 (6)
C7—H7⋯O1	0.95	2.35	2.920 (6)	118
C17—H17⋯O2	0.95	2.32	2.897 (5)	119
C17—H17⋯O32^i^	0.95	2.36	3.175 (7)	144
C22—H22⋯O12^ii^	0.95	2.50	3.108 (6)	122
C23—H23⋯O34^ii^	0.95	2.41	3.255 (7)	148
C34—H34*A*⋯O24	0.98	2.31	3.130 (10)	140
C34—H34*C*⋯O11^iii^	0.98	2.56	3.272 (9)	130
C36—H36*A*⋯O21^iv^	0.98	2.46	3.429 (8)	168
C38—H38*B*⋯N31^iv^	0.98	2.57	3.454 (10)	150
C40—H40*C*⋯O32^v^	0.98	2.57	3.516 (8)	162

Symmetry codes: (i) 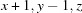; (ii) 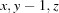; (iii) 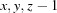; (iv) 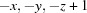; (v) 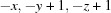.

**Table 5:** Experimental details

	**H_2_L1**	**I**
Crystal data		
Chemical formula	C_24_H_16_N_6_O_2_	[Cu_2_(C_24_H_15_N_6_O_2_)(C_2_H_3_N)_4_](ClO_4_)_3_·2C_2_H_3_N
*M*_r_	420.43	1091.17
Crystal system, space group	Triclinic, *P* 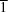	Triclinic, *P* 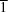
Temperature (K)	223	153
*a*, *b*, *c* (Å)	7.9633 (11), 8.0043 (12), 15.615 (2)	12.6281 (10), 13.4938 (11), 14.7884 (14)
α, β, γ (°)	97.629 (14), 98.349 (11), 100.407 (17)	74.678 (10), 89.115 (10), 65.170 (9)
*V* (Å^3^)	955.6 (2)	2192.5 (4)
*Z*	2	2
Radiation type	Cu *K*α	Mo *K*α
μ (mm^−1^)	0.80	1.23
Crystal size (mm)	0.46 × 0.23 × 0.15	0.30 × 0.30 × 0.15

Data collection		
Diffractometer	STOE-Siemens AED2, 4-circle	STOE *IPDS* 1
Absorption correction	Multi-scan (*MULABS*; Spek, 2020 ▸)	Multi-scan (*MULABS*; Spek, 2020 ▸)
*T*_min_, *T*_max_	0.984, 1.000	0.857, 1.000
No. of measured, independent and observed [*I* > 2σ(*I*)] reflections	5536, 2791, 2596	17344, 7942, 4757
*R*_int_	0.017	0.077
θ_max_ (°)	59.6	25.9
(sin θ/λ)_max_ (Å^−1^)	0.560	0.615

Refinement		
*R*[*F* ^2^ > 2σ(*F* ^2^)], *wR*(*F* ^2^), *S*	0.032, 0.092, 1.04	0.053, 0.138, 0.86
No. of reflections	2791	7942
No. of parameters	298	615
H-atom treatment	H atoms treated by a mixture of independent and constrained refinement	H atoms treated by a mixture of independent and constrained refinement
Δρ_max_, Δρ_min_ (e Å^−3^)	0.20, −0.17	0.91, −0.93

Computer programs: *STADI4* (Stoe & Cie, 1997 ▸), *EXPOSE*, *CELL* and *INTEGRATE* in *IPDS1* (Stoe & Cie, 2004 ▸), *X-RED* (Stoe & Cie, 1997 ▸), *SHELXS97* (Sheldrick, 2008 ▸), *SHELXL2014/6* and *SHELXL2016/6* (Sheldrick, 2015 ▸), *PLATON* (Spek, 2020 ▸), *Mercury* (Macrae *et al.*, 2020 ▸) and *publCIF* (Westrip, 2010 ▸).

## supplementary crystallographic information

### N2,N3-Bis(quinolin-8-yl)pyrazine-2,3-dicarboxamide (H2L1) Crystal data

**Table:**

C_24_H_16_N_6_O_2_	*Z* = 2
*M**_r_* = 420.43	*F*(000) = 436
Triclinic, *P*1	*D*_x_ = 1.461 Mg m^−^^3^
*a* = 7.9633 (11) Å	Cu *K*α radiation, λ = 1.54186 Å
*b* = 8.0043 (12) Å	Cell parameters from 21 reflections
*c* = 15.615 (2) Å	θ = 15.5–27.3°
α = 97.629 (14)°	µ = 0.80 mm^−^^1^
β = 98.349 (11)°	*T* = 223 K
γ = 100.407 (17)°	Rod, pale_yellow
*V* = 955.6 (2) Å^3^	0.46 × 0.23 × 0.15 mm

### N2,N3-Bis(quinolin-8-yl)pyrazine-2,3-dicarboxamide (H2L1) Data collection

**Table:**

STOE-Siemens AED2, 4-circle diffractometer	2596 reflections with *I* > 2σ(*I*)
Radiation source: fine-focus sealed tube	*R*_int_ = 0.017
Plane graphite monochromator	θ_max_ = 59.6°, θ_min_ = 2.9°
ω/2θ scans	*h* = −8→8
Absorption correction: multi-scan (MULABS; Spek, 2009)	*k* = −8→8
*T*_min_ = 0.984, *T*_max_ = 1.000	*l* = −17→17
5536 measured reflections	2 standard reflections every 60 min
2791 independent reflections	intensity decay: 2%

### N2,N3-Bis(quinolin-8-yl)pyrazine-2,3-dicarboxamide (H2L1) Refinement

**Table:**

Refinement on *F*^2^	Secondary atom site location: difference Fourier map
Least-squares matrix: full	Hydrogen site location: mixed
*R*[*F*^2^ > 2σ(*F*^2^)] = 0.032	H atoms treated by a mixture of independent and constrained refinement
*wR*(*F*^2^) = 0.092	*w* = 1/[σ^2^(*F*_o_^2^) + (0.0602*P*)^2^ + 0.1623*P*] where *P* = (*F*_o_^2^ + 2*F*_c_^2^)/3
*S* = 1.03	(Δ/σ)_max_ < 0.001
2791 reflections	Δρ_max_ = 0.20 e Å^−^^3^
298 parameters	Δρ_min_ = −0.17 e Å^−^^3^
0 restraints	Extinction correction: (SHELXL-2016/6; Sheldrick, 2015), Fc^*^=kFc[1+0.001xFc^2^λ^3^/sin(2θ)]^-1/4^
Primary atom site location: structure-invariant direct methods	Extinction coefficient: 0.0083 (8)

### N2,N3-Bis(quinolin-8-yl)pyrazine-2,3-dicarboxamide (H2L1) Special details

**Table:**

Geometry. All esds (except the esd in the dihedral angle between two l.s. planes) are estimated using the full covariance matrix. The cell esds are taken into account individually in the estimation of esds in distances, angles and torsion angles; correlations between esds in cell parameters are only used when they are defined by crystal symmetry. An approximate (isotropic) treatment of cell esds is used for estimating esds involving l.s. planes.

### N2,N3-Bis(quinolin-8-yl)pyrazine-2,3-dicarboxamide (H2L1) Fractional atomic coordinates and isotropic or equivalent isotropic displacement parameters (Å^2^)

**Table:**

	*x*	*y*	*z*	*U*_iso_*/*U*_eq_	
N1	0.03840 (15)	1.12688 (15)	0.36884 (8)	0.0351 (3)	
N2	−0.16596 (15)	1.03169 (15)	0.20236 (8)	0.0365 (3)	
N3	0.17902 (15)	0.89849 (16)	0.45078 (7)	0.0334 (3)	
H3N	0.200 (2)	1.011 (2)	0.4667 (10)	0.038 (4)*	
N4	0.38594 (14)	1.09265 (14)	0.59206 (7)	0.0317 (3)	
N5	−0.26526 (15)	0.64169 (15)	0.16840 (7)	0.0298 (3)	
H5N	−0.343 (2)	0.668 (2)	0.1963 (11)	0.039 (4)*	
N6	−0.59109 (14)	0.47298 (14)	0.15991 (7)	0.0307 (3)	
O1	0.02271 (14)	0.67866 (12)	0.34455 (7)	0.0449 (3)	
O2	0.01629 (13)	0.72082 (14)	0.15181 (7)	0.0454 (3)	
C1	0.00279 (16)	0.96459 (17)	0.32722 (8)	0.0291 (3)	
C2	−0.09398 (16)	0.91763 (17)	0.24276 (9)	0.0295 (3)	
C3	−0.13458 (19)	1.19183 (19)	0.24608 (10)	0.0389 (4)	
H3	−0.185777	1.274605	0.220628	0.047*	
C4	−0.03009 (19)	1.24052 (18)	0.32706 (10)	0.0385 (4)	
H4	−0.006243	1.356654	0.353677	0.046*	
C5	0.06931 (17)	0.83168 (17)	0.37497 (9)	0.0314 (3)	
C6	0.26615 (18)	0.81207 (17)	0.50976 (9)	0.0321 (3)	
C7	0.2510 (2)	0.63717 (19)	0.50045 (10)	0.0446 (4)	
H7	0.177771	0.566095	0.451485	0.054*	
C8	0.3440 (2)	0.5635 (2)	0.56341 (11)	0.0529 (5)	
H8	0.331749	0.443146	0.556124	0.063*	
C9	0.4514 (2)	0.6625 (2)	0.63477 (11)	0.0486 (4)	
H9	0.512816	0.610421	0.676168	0.058*	
C10	0.47108 (19)	0.84334 (18)	0.64690 (9)	0.0347 (3)	
C11	0.57800 (19)	0.95486 (19)	0.71977 (9)	0.0372 (4)	
H11	0.642381	0.909866	0.763226	0.045*	
C12	0.58759 (18)	1.12744 (19)	0.72692 (9)	0.0364 (3)	
H12	0.658863	1.203331	0.774906	0.044*	
C13	0.48898 (19)	1.18985 (18)	0.66126 (9)	0.0354 (3)	
H13	0.496900	1.309536	0.666969	0.043*	
C14	0.37686 (17)	0.91962 (17)	0.58438 (8)	0.0294 (3)	
C15	−0.10800 (17)	0.74740 (18)	0.18488 (8)	0.0304 (3)	
C16	−0.32246 (17)	0.49008 (16)	0.10646 (8)	0.0270 (3)	
C17	−0.22451 (18)	0.42460 (17)	0.04976 (9)	0.0311 (3)	
H17	−0.107818	0.478027	0.053540	0.037*	
C18	−0.29793 (19)	0.27840 (18)	−0.01373 (9)	0.0339 (3)	
H18	−0.229132	0.235331	−0.051940	0.041*	
C19	−0.46653 (19)	0.19748 (18)	−0.02128 (9)	0.0341 (3)	
H19	−0.513986	0.101611	−0.065281	0.041*	
C20	−0.56981 (17)	0.25848 (16)	0.03745 (8)	0.0297 (3)	
C21	−0.74570 (18)	0.18132 (18)	0.03417 (9)	0.0360 (4)	
H21	−0.798983	0.083571	−0.007714	0.043*	
C22	−0.83701 (19)	0.24976 (19)	0.09212 (10)	0.0374 (4)	
H22	−0.953714	0.199482	0.090878	0.045*	
C23	−0.75489 (18)	0.39644 (18)	0.15383 (9)	0.0350 (3)	
H23	−0.820165	0.442744	0.193010	0.042*	
C24	−0.49857 (17)	0.40505 (16)	0.10189 (8)	0.0262 (3)	

### N2,N3-Bis(quinolin-8-yl)pyrazine-2,3-dicarboxamide (H2L1) Atomic displacement parameters (Å^2^)

**Table:**

	*U*^11^	*U*^22^	*U*^33^	*U*^12^	*U*^13^	*U*^23^
N1	0.0356 (6)	0.0310 (7)	0.0333 (6)	0.0009 (5)	−0.0001 (5)	0.0007 (5)
N2	0.0365 (7)	0.0386 (7)	0.0330 (6)	0.0065 (5)	0.0003 (5)	0.0081 (5)
N3	0.0414 (7)	0.0272 (7)	0.0266 (6)	0.0034 (5)	−0.0045 (5)	0.0022 (5)
N4	0.0353 (7)	0.0290 (6)	0.0277 (6)	0.0045 (5)	0.0006 (5)	0.0022 (5)
N5	0.0260 (6)	0.0329 (6)	0.0275 (6)	0.0042 (5)	0.0027 (5)	−0.0012 (5)
N6	0.0311 (6)	0.0322 (6)	0.0288 (6)	0.0073 (5)	0.0036 (5)	0.0058 (5)
O1	0.0560 (7)	0.0293 (6)	0.0385 (6)	−0.0005 (5)	−0.0121 (5)	0.0013 (5)
O2	0.0286 (6)	0.0562 (7)	0.0446 (6)	0.0027 (5)	0.0065 (5)	−0.0079 (5)
C1	0.0264 (7)	0.0301 (7)	0.0268 (7)	−0.0012 (5)	0.0024 (5)	0.0022 (6)
C2	0.0237 (7)	0.0343 (7)	0.0287 (7)	0.0010 (5)	0.0035 (5)	0.0070 (6)
C3	0.0418 (8)	0.0353 (8)	0.0397 (8)	0.0077 (6)	0.0032 (7)	0.0110 (7)
C4	0.0419 (8)	0.0283 (8)	0.0421 (8)	0.0035 (6)	0.0031 (7)	0.0039 (6)
C5	0.0327 (7)	0.0301 (8)	0.0271 (7)	−0.0001 (6)	0.0011 (6)	0.0022 (6)
C6	0.0374 (8)	0.0311 (8)	0.0250 (7)	0.0043 (6)	0.0004 (6)	0.0036 (6)
C7	0.0574 (10)	0.0325 (8)	0.0350 (8)	0.0033 (7)	−0.0095 (7)	0.0011 (6)
C8	0.0745 (12)	0.0291 (8)	0.0466 (9)	0.0086 (8)	−0.0125 (9)	0.0038 (7)
C9	0.0643 (11)	0.0352 (8)	0.0417 (9)	0.0136 (8)	−0.0116 (8)	0.0079 (7)
C10	0.0377 (8)	0.0355 (8)	0.0290 (7)	0.0072 (6)	0.0006 (6)	0.0050 (6)
C11	0.0371 (8)	0.0424 (9)	0.0298 (8)	0.0095 (6)	−0.0032 (6)	0.0054 (6)
C12	0.0335 (8)	0.0401 (8)	0.0292 (7)	0.0022 (6)	−0.0019 (6)	−0.0026 (6)
C13	0.0391 (8)	0.0309 (8)	0.0320 (8)	0.0034 (6)	0.0021 (6)	−0.0005 (6)
C14	0.0309 (7)	0.0303 (7)	0.0258 (7)	0.0043 (6)	0.0046 (6)	0.0032 (5)
C15	0.0265 (7)	0.0378 (8)	0.0241 (7)	0.0045 (6)	−0.0014 (5)	0.0045 (6)
C16	0.0294 (7)	0.0281 (7)	0.0225 (6)	0.0069 (5)	−0.0007 (5)	0.0047 (5)
C17	0.0304 (7)	0.0343 (7)	0.0301 (7)	0.0099 (6)	0.0036 (6)	0.0071 (6)
C18	0.0432 (8)	0.0334 (8)	0.0283 (7)	0.0150 (6)	0.0078 (6)	0.0047 (6)
C19	0.0454 (9)	0.0278 (7)	0.0283 (7)	0.0101 (6)	0.0018 (6)	0.0031 (6)
C20	0.0358 (8)	0.0256 (7)	0.0270 (7)	0.0076 (6)	−0.0014 (6)	0.0070 (5)
C21	0.0379 (8)	0.0282 (7)	0.0364 (8)	0.0015 (6)	−0.0041 (6)	0.0050 (6)
C22	0.0299 (7)	0.0372 (8)	0.0426 (8)	0.0017 (6)	0.0008 (6)	0.0107 (7)
C23	0.0304 (8)	0.0377 (8)	0.0384 (8)	0.0073 (6)	0.0066 (6)	0.0099 (6)
C24	0.0293 (7)	0.0257 (7)	0.0239 (7)	0.0080 (5)	0.0002 (5)	0.0068 (5)

### N2,N3-Bis(quinolin-8-yl)pyrazine-2,3-dicarboxamide (H2L1) Geometric parameters (Å, º)

**Table:**

N1—C1	1.3339 (18)	C8—C9	1.359 (2)
N1—C4	1.3358 (19)	C8—H8	0.9400
N2—C3	1.3319 (19)	C9—C10	1.412 (2)
N2—C2	1.3406 (18)	C9—H9	0.9400
N3—C5	1.3466 (18)	C10—C11	1.411 (2)
N3—C6	1.4018 (18)	C10—C14	1.414 (2)
N3—H3N	0.880 (17)	C11—C12	1.358 (2)
N4—C13	1.3171 (18)	C11—H11	0.9400
N4—C14	1.3620 (18)	C12—C13	1.401 (2)
N5—C15	1.3477 (18)	C12—H12	0.9400
N5—C16	1.4058 (17)	C13—H13	0.9400
N5—H5N	0.846 (17)	C16—C17	1.3756 (19)
N6—C23	1.3212 (18)	C16—C24	1.4312 (19)
N6—C24	1.3663 (18)	C17—C18	1.404 (2)
O1—C5	1.2222 (17)	C17—H17	0.9400
O2—C15	1.2193 (16)	C18—C19	1.362 (2)
C1—C2	1.3940 (19)	C18—H18	0.9400
C1—C5	1.506 (2)	C19—C20	1.415 (2)
C2—C15	1.5082 (19)	C19—H19	0.9400
C3—C4	1.375 (2)	C20—C24	1.4111 (19)
C3—H3	0.9400	C20—C21	1.416 (2)
C4—H4	0.9400	C21—C22	1.360 (2)
C6—C7	1.369 (2)	C21—H21	0.9400
C6—C14	1.4273 (19)	C22—C23	1.405 (2)
C7—C8	1.401 (2)	C22—H22	0.9400
C7—H7	0.9400	C23—H23	0.9400
			
C1—N1—C4	116.56 (12)	C12—C11—H11	120.1
C3—N2—C2	116.33 (12)	C10—C11—H11	120.1
C5—N3—C6	128.53 (12)	C11—C12—C13	118.55 (13)
C5—N3—H3N	117.3 (10)	C11—C12—H12	120.7
C6—N3—H3N	114.2 (10)	C13—C12—H12	120.7
C13—N4—C14	116.99 (12)	N4—C13—C12	124.61 (13)
C15—N5—C16	127.71 (12)	N4—C13—H13	117.7
C15—N5—H5N	118.6 (11)	C12—C13—H13	117.7
C16—N5—H5N	113.7 (11)	N4—C14—C10	122.93 (12)
C23—N6—C24	117.39 (12)	N4—C14—C6	117.86 (12)
N1—C1—C2	121.70 (13)	C10—C14—C6	119.21 (12)
N1—C1—C5	117.72 (12)	O2—C15—N5	125.13 (13)
C2—C1—C5	120.58 (12)	O2—C15—C2	118.87 (12)
N2—C2—C1	121.15 (13)	N5—C15—C2	115.76 (12)
N2—C2—C15	114.00 (11)	C17—C16—N5	124.89 (12)
C1—C2—C15	124.37 (12)	C17—C16—C24	119.45 (12)
N2—C3—C4	122.43 (14)	N5—C16—C24	115.61 (12)
N2—C3—H3	118.8	C16—C17—C18	120.33 (13)
C4—C3—H3	118.8	C16—C17—H17	119.8
N1—C4—C3	121.62 (14)	C18—C17—H17	119.8
N1—C4—H4	119.2	C19—C18—C17	121.56 (13)
C3—C4—H4	119.2	C19—C18—H18	119.2
O1—C5—N3	125.78 (13)	C17—C18—H18	119.2
O1—C5—C1	120.32 (12)	C18—C19—C20	119.65 (13)
N3—C5—C1	113.91 (12)	C18—C19—H19	120.2
C7—C6—N3	124.96 (13)	C20—C19—H19	120.2
C7—C6—C14	119.65 (13)	C24—C20—C21	117.14 (13)
N3—C6—C14	115.39 (12)	C24—C20—C19	119.73 (12)
C6—C7—C8	120.45 (14)	C21—C20—C19	123.12 (13)
C6—C7—H7	119.8	C22—C21—C20	119.48 (13)
C8—C7—H7	119.8	C22—C21—H21	120.3
C9—C8—C7	121.29 (15)	C20—C21—H21	120.3
C9—C8—H8	119.4	C21—C22—C23	119.24 (13)
C7—C8—H8	119.4	C21—C22—H22	120.4
C8—C9—C10	120.14 (14)	C23—C22—H22	120.4
C8—C9—H9	119.9	N6—C23—C22	123.70 (13)
C10—C9—H9	119.9	N6—C23—H23	118.1
C11—C10—C9	123.61 (13)	C22—C23—H23	118.1
C11—C10—C14	117.12 (13)	N6—C24—C20	123.04 (12)
C9—C10—C14	119.26 (13)	N6—C24—C16	117.72 (12)
C12—C11—C10	119.79 (13)	C20—C24—C16	119.23 (12)
			
C4—N1—C1—C2	3.0 (2)	C9—C10—C14—C6	−1.0 (2)
C4—N1—C1—C5	−176.41 (12)	C7—C6—C14—N4	−178.83 (13)
C3—N2—C2—C1	2.27 (19)	N3—C6—C14—N4	0.86 (19)
C3—N2—C2—C15	−170.17 (12)	C7—C6—C14—C10	0.9 (2)
N1—C1—C2—N2	−4.9 (2)	N3—C6—C14—C10	−179.42 (12)
C5—C1—C2—N2	174.45 (12)	C16—N5—C15—O2	−5.5 (2)
N1—C1—C2—C15	166.71 (12)	C16—N5—C15—C2	168.88 (12)
C5—C1—C2—C15	−13.9 (2)	N2—C2—C15—O2	99.14 (15)
C2—N2—C3—C4	1.9 (2)	C1—C2—C15—O2	−73.02 (18)
C1—N1—C4—C3	1.2 (2)	N2—C2—C15—N5	−75.58 (15)
N2—C3—C4—N1	−3.8 (2)	C1—C2—C15—N5	112.26 (14)
C6—N3—C5—O1	1.6 (2)	C15—N5—C16—C17	−1.2 (2)
C6—N3—C5—C1	−178.64 (13)	C15—N5—C16—C24	−178.69 (12)
N1—C1—C5—O1	170.92 (13)	N5—C16—C17—C18	−175.64 (12)
C2—C1—C5—O1	−8.5 (2)	C24—C16—C17—C18	1.78 (19)
N1—C1—C5—N3	−8.81 (18)	C16—C17—C18—C19	0.0 (2)
C2—C1—C5—N3	171.79 (12)	C17—C18—C19—C20	−1.7 (2)
C5—N3—C6—C7	−1.3 (2)	C18—C19—C20—C24	1.56 (19)
C5—N3—C6—C14	179.02 (13)	C18—C19—C20—C21	−179.78 (12)
N3—C6—C7—C8	−179.94 (15)	C24—C20—C21—C22	−0.14 (19)
C14—C6—C7—C8	−0.3 (2)	C19—C20—C21—C22	−178.84 (12)
C6—C7—C8—C9	−0.2 (3)	C20—C21—C22—C23	0.3 (2)
C7—C8—C9—C10	0.1 (3)	C24—N6—C23—C22	0.6 (2)
C8—C9—C10—C11	179.05 (16)	C21—C22—C23—N6	−0.6 (2)
C8—C9—C10—C14	0.5 (3)	C23—N6—C24—C20	−0.43 (18)
C9—C10—C11—C12	−179.09 (15)	C23—N6—C24—C16	178.29 (11)
C14—C10—C11—C12	−0.5 (2)	C21—C20—C24—N6	0.19 (18)
C10—C11—C12—C13	0.4 (2)	C19—C20—C24—N6	178.93 (11)
C14—N4—C13—C12	−0.5 (2)	C21—C20—C24—C16	−178.51 (11)
C11—C12—C13—N4	0.1 (2)	C19—C20—C24—C16	0.23 (18)
C13—N4—C14—C10	0.4 (2)	C17—C16—C24—N6	179.34 (11)
C13—N4—C14—C6	−179.89 (12)	N5—C16—C24—N6	−3.01 (17)
C11—C10—C14—N4	0.1 (2)	C17—C16—C24—C20	−1.88 (18)
C9—C10—C14—N4	178.73 (14)	N5—C16—C24—C20	175.77 (11)
C11—C10—C14—C6	−179.65 (12)		

### N2,N3-Bis(quinolin-8-yl)pyrazine-2,3-dicarboxamide (H2L1) Hydrogen-bond geometry (Å, º)

**Table:**

*D*—H···*A*	*D*—H	H···*A*	*D*···*A*	*D*—H···*A*
N3—H3*N*···N1	0.88 (2)	2.256 (16)	2.6791 (18)	109 (1)
N3—H3*N*···N4	0.88 (2)	2.218 (16)	2.6657 (16)	111 (1)
N5—H5*N*···N6	0.85 (2)	2.233 (16)	2.6759 (17)	113 (1)
C7—H7···O1	0.94	2.31	2.9136 (19)	122
C17—H17···O2	0.94	2.28	2.8818 (18)	122
C4—H4···O1^i^	0.94	2.57	3.4249 (18)	151
C12—H12···O2^ii^	0.94	2.60	3.3589 (19)	138
C18—H18···O2^iii^	0.94	2.48	3.3289 (19)	151

Symmetry codes: (i) *x*, *y*+1, *z*; (ii) −*x*+1, −*y*+2, −*z*+1; (iii) −*x*, −*y*+1, −*z*.

### [µ-(3-{Hydroxy[(quinolin-8-yl)imino]methyl}pyrazin-2-yl)[(quinolin-8-yl)imino]methanolato]bis[diacetonitrilecopper(II)] tris(perchlorate) acetonitrile disolvate (I) Crystal data

**Table:**

[Cu_2_(C_24_H_15_N_6_O_2_)(C_2_H_3_N)_4_](ClO_4_)_3_·2C_2_H_3_N	*Z* = 2
*M**_r_* = 1091.17	*F*(000) = 1108
Triclinic, *P*1	*D*_x_ = 1.653 Mg m^−^^3^
*a* = 12.6281 (10) Å	Mo *K*α radiation, λ = 0.71073 Å
*b* = 13.4938 (11) Å	Cell parameters from 8000 reflections
*c* = 14.7884 (14) Å	θ = 1.9–25.9°
α = 74.678 (10)°	µ = 1.23 mm^−^^1^
β = 89.115 (10)°	*T* = 153 K
γ = 65.170 (9)°	Plate, green
*V* = 2192.5 (4) Å^3^	0.30 × 0.30 × 0.15 mm

### [µ-(3-{Hydroxy[(quinolin-8-yl)imino]methyl}pyrazin-2-yl)[(quinolin-8-yl)imino]methanolato]bis[diacetonitrilecopper(II)] tris(perchlorate) acetonitrile disolvate (I) Data collection

**Table:**

STOE IPDS 1 diffractometer	7942 independent reflections
Radiation source: fine-focus sealed tube	4757 reflections with *I* > 2σ(*I*)
Plane graphite monochromator	*R*_int_ = 0.077
φ rotation scans	θ_max_ = 25.9°, θ_min_ = 1.9°
Absorption correction: multi-scan (MULABS; Spek, 2009)	*h* = −15→15
*T*_min_ = 0.857, *T*_max_ = 1.000	*k* = −16→16
17344 measured reflections	*l* = −18→18

### [µ-(3-{Hydroxy[(quinolin-8-yl)imino]methyl}pyrazin-2-yl)[(quinolin-8-yl)imino]methanolato]bis[diacetonitrilecopper(II)] tris(perchlorate) acetonitrile disolvate (I) Refinement

**Table:**

Refinement on *F*^2^	Secondary atom site location: difference Fourier map
Least-squares matrix: full	Hydrogen site location: mixed
*R*[*F*^2^ > 2σ(*F*^2^)] = 0.053	H atoms treated by a mixture of independent and constrained refinement
*wR*(*F*^2^) = 0.138	*w* = 1/[σ^2^(*F*_o_^2^) + (0.0806*P*)^2^] where *P* = (*F*_o_^2^ + 2*F*_c_^2^)/3
*S* = 0.86	(Δ/σ)_max_ < 0.001
7942 reflections	Δρ_max_ = 0.91 e Å^−^^3^
615 parameters	Δρ_min_ = −0.93 e Å^−^^3^
0 restraints	Extinction correction: (SHELXL-2016/6; Sheldrick, 2015), Fc^*^=kFc[1+0.001xFc^2^λ^3^/sin(2θ)]^-1/4^
Primary atom site location: structure-invariant direct methods	Extinction coefficient: 0.0021 (6)

### [µ-(3-{Hydroxy[(quinolin-8-yl)imino]methyl}pyrazin-2-yl)[(quinolin-8-yl)imino]methanolato]bis[diacetonitrilecopper(II)] tris(perchlorate) acetonitrile disolvate (I) Special details

**Table:**

Geometry. All esds (except the esd in the dihedral angle between two l.s. planes) are estimated using the full covariance matrix. The cell esds are taken into account individually in the estimation of esds in distances, angles and torsion angles; correlations between esds in cell parameters are only used when they are defined by crystal symmetry. An approximate (isotropic) treatment of cell esds is used for estimating esds involving l.s. planes.

### [µ-(3-{Hydroxy[(quinolin-8-yl)imino]methyl}pyrazin-2-yl)[(quinolin-8-yl)imino]methanolato]bis[diacetonitrilecopper(II)] tris(perchlorate) acetonitrile disolvate (I) Fractional atomic coordinates and isotropic or equivalent isotropic displacement parameters (Å^2^)

**Table:**

	*x*	*y*	*z*	*U*_iso_*/*U*_eq_	
Cu1	0.31089 (4)	0.36323 (4)	0.17677 (4)	0.02595 (17)	
Cu2	0.33585 (5)	−0.14019 (4)	0.43021 (4)	0.02764 (17)	
N1	0.3033 (3)	0.2198 (3)	0.2544 (2)	0.0240 (8)	
N2	0.3161 (3)	0.0140 (3)	0.3545 (2)	0.0266 (9)	
N3	0.4715 (3)	0.2794 (3)	0.2351 (2)	0.0260 (9)	
N4	0.3613 (3)	0.4827 (3)	0.1225 (2)	0.0262 (9)	
N5	0.4906 (3)	−0.1517 (3)	0.4603 (2)	0.0234 (8)	
N6	0.4004 (3)	−0.2963 (3)	0.5139 (2)	0.0256 (9)	
O1	0.6081 (3)	0.1110 (3)	0.3337 (2)	0.0307 (8)	
H2O	0.622 (5)	0.012 (5)	0.386 (4)	0.059 (17)*	
O2	0.6151 (3)	−0.0656 (3)	0.4298 (2)	0.0281 (7)	
C1	0.4089 (4)	0.1378 (3)	0.3039 (3)	0.0225 (10)	
C2	0.4127 (4)	0.0330 (4)	0.3559 (3)	0.0239 (10)	
C3	0.2154 (4)	0.0958 (4)	0.3057 (3)	0.0288 (11)	
H3	0.147651	0.081917	0.305320	0.035*	
C4	0.2096 (4)	0.2002 (4)	0.2561 (3)	0.0257 (10)	
H4	0.137294	0.258614	0.222702	0.031*	
C5	0.5038 (4)	0.1753 (4)	0.2911 (3)	0.0250 (10)	
C6	0.5416 (4)	0.3376 (4)	0.2101 (3)	0.0247 (10)	
C7	0.6599 (4)	0.3015 (4)	0.2368 (3)	0.0290 (11)	
H7	0.704730	0.226789	0.276687	0.035*	
C8	0.7137 (4)	0.3756 (4)	0.2047 (3)	0.0323 (12)	
H8	0.794450	0.350005	0.224037	0.039*	
C9	0.6518 (4)	0.4835 (4)	0.1464 (3)	0.0309 (11)	
H9	0.689483	0.532160	0.125900	0.037*	
C10	0.5319 (4)	0.5223 (4)	0.1169 (3)	0.0269 (11)	
C11	0.4626 (4)	0.6320 (4)	0.0526 (3)	0.0323 (11)	
H11	0.496049	0.683626	0.028325	0.039*	
C12	0.3481 (4)	0.6609 (4)	0.0269 (3)	0.0318 (12)	
H12	0.301292	0.733390	−0.015634	0.038*	
C13	0.2994 (4)	0.5859 (4)	0.0622 (3)	0.0278 (11)	
H13	0.219212	0.608312	0.043100	0.033*	
C14	0.4778 (4)	0.4509 (4)	0.1486 (3)	0.0229 (10)	
C15	0.5170 (4)	−0.0680 (4)	0.4180 (3)	0.0225 (10)	
C16	0.5693 (4)	−0.2589 (4)	0.5187 (3)	0.0257 (11)	
C17	0.6844 (4)	−0.2951 (4)	0.5505 (3)	0.0270 (10)	
H17	0.721286	−0.245362	0.532594	0.032*	
C18	0.7484 (4)	−0.4074 (4)	0.6102 (3)	0.0331 (12)	
H18	0.828775	−0.432457	0.630637	0.040*	
C19	0.6979 (4)	−0.4800 (4)	0.6390 (3)	0.0321 (12)	
H19	0.742703	−0.554228	0.679823	0.038*	
C20	0.5791 (4)	−0.4457 (4)	0.6086 (3)	0.0284 (11)	
C21	0.5187 (4)	−0.5154 (4)	0.6354 (3)	0.0330 (12)	
H21	0.557824	−0.590133	0.676896	0.040*	
C22	0.4040 (4)	−0.4741 (4)	0.6009 (3)	0.0333 (12)	
H22	0.362917	−0.520124	0.617845	0.040*	
C23	0.3473 (4)	−0.3628 (4)	0.5402 (3)	0.0318 (12)	
H23	0.267137	−0.334616	0.517247	0.038*	
C24	0.5152 (4)	−0.3350 (3)	0.5481 (3)	0.0237 (10)	
N11	0.1393 (3)	0.4596 (3)	0.1516 (3)	0.0312 (10)	
N12	0.3124 (4)	0.2964 (4)	0.0511 (3)	0.0339 (10)	
N21	0.1935 (4)	−0.0653 (3)	0.5072 (3)	0.0350 (10)	
N22	0.2413 (4)	−0.1562 (3)	0.3276 (3)	0.0340 (10)	
N31	−0.0216 (5)	0.3087 (5)	0.7312 (4)	0.0761 (19)	
N41	0.6128 (5)	0.1094 (4)	0.1045 (4)	0.0633 (15)	
C31	0.0403 (4)	0.5107 (4)	0.1374 (3)	0.0338 (12)	
C32	−0.0859 (4)	0.5739 (5)	0.1177 (4)	0.0529 (17)	
H32A	−0.106839	0.655648	0.097208	0.079*	
H32B	−0.122554	0.556263	0.174940	0.079*	
H32C	−0.113802	0.552464	0.067735	0.079*	
C33	0.3372 (4)	0.2123 (5)	0.0361 (3)	0.0352 (12)	
C34	0.3688 (6)	0.1031 (5)	0.0199 (5)	0.067 (2)	
H34A	0.300051	0.086608	0.022887	0.101*	
H34B	0.431395	0.043918	0.068279	0.101*	
H34C	0.396348	0.104566	−0.042546	0.101*	
C35	0.1362 (4)	−0.0221 (4)	0.5572 (3)	0.0361 (13)	
C36	0.0637 (5)	0.0322 (5)	0.6241 (4)	0.0548 (16)	
H36A	0.061187	−0.026485	0.678574	0.082*	
H36B	−0.016047	0.082587	0.593230	0.082*	
H36C	0.097525	0.076747	0.645207	0.082*	
C37	0.2107 (4)	−0.1557 (4)	0.2554 (3)	0.0329 (12)	
C38	0.1728 (6)	−0.1549 (5)	0.1627 (4)	0.0549 (17)	
H38A	0.118838	−0.076994	0.127438	0.082*	
H38B	0.132646	−0.204398	0.169850	0.082*	
H38C	0.241295	−0.182614	0.128469	0.082*	
C39	0.0411 (6)	0.3305 (5)	0.6832 (4)	0.0534 (16)	
C40	0.1219 (6)	0.3564 (6)	0.6225 (5)	0.0639 (18)	
H40A	0.201931	0.312437	0.654576	0.096*	
H40B	0.101443	0.437947	0.607678	0.096*	
H40C	0.117327	0.336521	0.564073	0.096*	
C41	0.7059 (6)	0.0440 (5)	0.1161 (4)	0.0468 (15)	
C42	0.8281 (8)	−0.0358 (9)	0.1254 (7)	0.134 (5)	
H42A	0.846983	−0.055433	0.066062	0.202*	
H42B	0.842543	−0.104882	0.176330	0.202*	
H42C	0.877582	−0.001087	0.140095	0.202*	
Cl1	0.45230 (12)	0.20687 (10)	0.77805 (8)	0.0413 (3)	
O11	0.3457 (5)	0.2730 (5)	0.8096 (3)	0.0921 (19)	
O12	0.4683 (5)	0.2688 (3)	0.6886 (3)	0.0808 (17)	
O13	0.5461 (5)	0.1804 (5)	0.8468 (3)	0.0925 (18)	
O14	0.4542 (5)	0.1041 (4)	0.7714 (3)	0.0713 (14)	
Cl2	0.00354 (11)	0.19052 (11)	0.08660 (8)	0.0419 (3)	
O21	−0.0275 (6)	0.1401 (5)	0.1734 (4)	0.102 (2)	
O22	−0.0253 (4)	0.3067 (3)	0.0811 (3)	0.0647 (12)	
O23	−0.0508 (5)	0.1810 (5)	0.0102 (4)	0.103 (2)	
O24	0.1275 (4)	0.1280 (5)	0.0930 (4)	0.098 (2)	
Cl3	0.01801 (11)	0.70326 (11)	0.59390 (8)	0.0390 (3)	
O31	0.0200 (4)	0.6207 (3)	0.6788 (3)	0.0688 (14)	
O32	−0.1024 (3)	0.7712 (4)	0.5514 (3)	0.0599 (12)	
O33	0.0616 (4)	0.7765 (3)	0.6140 (3)	0.0602 (12)	
O34	0.0865 (4)	0.6450 (4)	0.5308 (3)	0.0665 (13)	

### [µ-(3-{Hydroxy[(quinolin-8-yl)imino]methyl}pyrazin-2-yl)[(quinolin-8-yl)imino]methanolato]bis[diacetonitrilecopper(II)] tris(perchlorate) acetonitrile disolvate (I) Atomic displacement parameters (Å^2^)

**Table:**

	*U*^11^	*U*^22^	*U*^33^	*U*^12^	*U*^13^	*U*^23^
Cu1	0.0182 (3)	0.0212 (3)	0.0263 (3)	−0.0047 (2)	−0.0075 (2)	0.0066 (2)
Cu2	0.0211 (3)	0.0211 (3)	0.0300 (3)	−0.0063 (2)	−0.0075 (2)	0.0056 (2)
N1	0.0194 (18)	0.0234 (18)	0.0213 (18)	−0.0075 (16)	−0.0068 (14)	0.0039 (14)
N2	0.0217 (19)	0.0214 (19)	0.0269 (19)	−0.0057 (16)	−0.0065 (15)	0.0028 (15)
N3	0.0241 (19)	0.0215 (19)	0.0194 (17)	−0.0047 (17)	−0.0066 (14)	0.0065 (14)
N4	0.0212 (19)	0.0223 (19)	0.0246 (18)	−0.0040 (16)	−0.0066 (15)	0.0015 (15)
N5	0.0202 (18)	0.0188 (18)	0.0224 (18)	−0.0048 (16)	−0.0067 (14)	0.0024 (14)
N6	0.0238 (19)	0.0208 (18)	0.0249 (18)	−0.0078 (17)	−0.0027 (15)	0.0024 (15)
O1	0.0190 (15)	0.0286 (17)	0.0311 (16)	−0.0083 (14)	−0.0107 (13)	0.0102 (13)
O2	0.0196 (16)	0.0237 (16)	0.0296 (16)	−0.0069 (14)	−0.0111 (13)	0.0070 (13)
C1	0.018 (2)	0.020 (2)	0.020 (2)	−0.0019 (18)	−0.0079 (16)	−0.0026 (17)
C2	0.023 (2)	0.021 (2)	0.022 (2)	−0.0066 (19)	−0.0043 (17)	−0.0011 (17)
C3	0.016 (2)	0.027 (2)	0.034 (2)	−0.0056 (19)	−0.0082 (18)	0.0013 (19)
C4	0.021 (2)	0.023 (2)	0.024 (2)	−0.0072 (19)	−0.0062 (17)	0.0040 (18)
C5	0.021 (2)	0.027 (2)	0.022 (2)	−0.008 (2)	−0.0026 (17)	−0.0036 (18)
C6	0.022 (2)	0.021 (2)	0.022 (2)	−0.0059 (19)	−0.0049 (17)	0.0013 (17)
C7	0.026 (2)	0.029 (2)	0.025 (2)	−0.010 (2)	−0.0063 (18)	0.0007 (19)
C8	0.025 (2)	0.031 (3)	0.031 (2)	−0.011 (2)	−0.0079 (19)	0.005 (2)
C9	0.030 (3)	0.030 (2)	0.032 (2)	−0.016 (2)	0.000 (2)	−0.002 (2)
C10	0.028 (2)	0.022 (2)	0.022 (2)	−0.006 (2)	−0.0031 (18)	0.0007 (17)
C11	0.038 (3)	0.022 (2)	0.032 (2)	−0.013 (2)	0.003 (2)	0.0006 (19)
C12	0.030 (3)	0.020 (2)	0.031 (2)	−0.003 (2)	−0.005 (2)	0.0037 (19)
C13	0.021 (2)	0.022 (2)	0.029 (2)	−0.0048 (19)	−0.0066 (18)	0.0034 (18)
C14	0.023 (2)	0.022 (2)	0.018 (2)	−0.0070 (19)	−0.0030 (17)	−0.0014 (17)
C15	0.019 (2)	0.022 (2)	0.020 (2)	−0.0066 (19)	−0.0054 (17)	0.0003 (17)
C16	0.024 (2)	0.024 (2)	0.018 (2)	−0.0016 (19)	−0.0036 (17)	−0.0017 (17)
C17	0.026 (2)	0.025 (2)	0.022 (2)	−0.009 (2)	−0.0028 (18)	0.0015 (18)
C18	0.028 (2)	0.029 (2)	0.026 (2)	−0.002 (2)	−0.0086 (19)	0.0031 (19)
C19	0.031 (3)	0.026 (2)	0.024 (2)	−0.006 (2)	−0.0081 (19)	0.0067 (19)
C20	0.031 (2)	0.022 (2)	0.023 (2)	−0.006 (2)	−0.0026 (18)	0.0008 (18)
C21	0.034 (3)	0.020 (2)	0.032 (2)	−0.006 (2)	−0.004 (2)	0.0022 (19)
C22	0.038 (3)	0.026 (2)	0.032 (2)	−0.015 (2)	0.003 (2)	0.0009 (19)
C23	0.025 (2)	0.026 (2)	0.037 (3)	−0.007 (2)	−0.003 (2)	−0.002 (2)
C24	0.024 (2)	0.018 (2)	0.022 (2)	−0.0053 (19)	−0.0020 (17)	0.0007 (17)
N11	0.025 (2)	0.024 (2)	0.031 (2)	−0.0047 (18)	−0.0053 (16)	0.0042 (16)
N12	0.034 (2)	0.034 (2)	0.028 (2)	−0.015 (2)	−0.0069 (17)	0.0001 (18)
N21	0.027 (2)	0.030 (2)	0.032 (2)	−0.0031 (18)	−0.0054 (18)	0.0013 (18)
N22	0.033 (2)	0.027 (2)	0.035 (2)	−0.0111 (18)	−0.0042 (18)	0.0000 (17)
N31	0.062 (4)	0.070 (4)	0.063 (4)	−0.013 (3)	−0.006 (3)	0.009 (3)
N41	0.063 (4)	0.045 (3)	0.070 (4)	−0.016 (3)	0.003 (3)	−0.011 (3)
C31	0.026 (3)	0.031 (3)	0.029 (2)	−0.007 (2)	−0.0054 (19)	0.006 (2)
C32	0.021 (3)	0.059 (4)	0.051 (3)	−0.002 (3)	−0.011 (2)	0.004 (3)
C33	0.029 (3)	0.035 (3)	0.029 (2)	−0.009 (2)	−0.004 (2)	0.006 (2)
C34	0.072 (5)	0.045 (4)	0.079 (5)	−0.016 (3)	0.011 (4)	−0.024 (3)
C35	0.029 (3)	0.034 (3)	0.031 (3)	−0.009 (2)	−0.007 (2)	0.004 (2)
C36	0.051 (4)	0.059 (4)	0.046 (3)	−0.015 (3)	0.010 (3)	−0.017 (3)
C37	0.024 (2)	0.032 (3)	0.034 (3)	−0.007 (2)	−0.003 (2)	−0.005 (2)
C38	0.060 (4)	0.056 (4)	0.037 (3)	−0.013 (3)	−0.010 (3)	−0.014 (3)
C39	0.049 (4)	0.047 (3)	0.049 (4)	−0.012 (3)	−0.006 (3)	−0.005 (3)
C40	0.068 (4)	0.074 (4)	0.059 (4)	−0.036 (4)	0.010 (3)	−0.025 (3)
C41	0.056 (4)	0.035 (3)	0.038 (3)	−0.013 (3)	−0.008 (3)	−0.003 (2)
C42	0.075 (6)	0.122 (8)	0.155 (9)	0.027 (6)	−0.056 (6)	−0.072 (7)
Cl1	0.0529 (8)	0.0322 (6)	0.0319 (6)	−0.0135 (6)	0.0035 (5)	−0.0061 (5)
O11	0.072 (3)	0.086 (4)	0.071 (3)	0.012 (3)	0.012 (3)	−0.025 (3)
O12	0.160 (5)	0.043 (2)	0.042 (2)	−0.051 (3)	0.030 (3)	−0.0075 (19)
O13	0.084 (4)	0.131 (5)	0.067 (3)	−0.048 (4)	−0.012 (3)	−0.031 (3)
O14	0.130 (4)	0.054 (3)	0.050 (2)	−0.056 (3)	0.023 (3)	−0.019 (2)
Cl2	0.0319 (6)	0.0409 (7)	0.0377 (7)	−0.0044 (6)	−0.0097 (5)	−0.0054 (5)
O21	0.130 (5)	0.091 (4)	0.081 (4)	−0.055 (4)	0.039 (3)	−0.007 (3)
O22	0.062 (3)	0.040 (2)	0.078 (3)	−0.014 (2)	−0.007 (2)	−0.008 (2)
O23	0.087 (4)	0.102 (4)	0.084 (3)	0.005 (3)	−0.053 (3)	−0.043 (3)
O24	0.033 (2)	0.091 (4)	0.147 (5)	0.009 (3)	−0.023 (3)	−0.058 (4)
Cl3	0.0301 (6)	0.0420 (7)	0.0404 (7)	−0.0161 (6)	−0.0015 (5)	−0.0034 (5)
O31	0.083 (3)	0.045 (2)	0.061 (3)	−0.026 (2)	0.017 (2)	0.009 (2)
O32	0.0272 (19)	0.068 (3)	0.076 (3)	−0.013 (2)	−0.0099 (18)	−0.018 (2)
O33	0.058 (3)	0.051 (2)	0.071 (3)	−0.030 (2)	−0.025 (2)	−0.003 (2)
O34	0.044 (2)	0.094 (4)	0.063 (3)	−0.024 (2)	0.017 (2)	−0.036 (3)

### [µ-(3-{Hydroxy[(quinolin-8-yl)imino]methyl}pyrazin-2-yl)[(quinolin-8-yl)imino]methanolato]bis[diacetonitrilecopper(II)] tris(perchlorate) acetonitrile disolvate (I) Geometric parameters (Å, º)

**Table:**

Cu1—N1	2.013 (4)	C18—H18	0.9500
Cu1—N3	1.936 (3)	C19—C20	1.412 (7)
Cu1—N4	1.952 (4)	C19—H19	0.9500
Cu1—N11	1.982 (4)	C20—C24	1.409 (6)
Cu1—N12	2.266 (4)	C20—C21	1.422 (7)
Cu2—N2	1.996 (4)	C21—C22	1.364 (7)
Cu2—N5	1.943 (4)	C21—H21	0.9500
Cu2—N6	1.961 (3)	C22—C23	1.407 (6)
Cu2—N21	2.137 (4)	C22—H22	0.9500
Cu2—N22	2.054 (4)	C23—H23	0.9500
N1—C4	1.315 (6)	N11—C31	1.136 (6)
N1—C1	1.378 (5)	N12—C33	1.126 (6)
N2—C3	1.336 (5)	N21—C35	1.126 (6)
N2—C2	1.349 (6)	N22—C37	1.138 (6)
N3—C5	1.319 (6)	N31—C39	1.133 (8)
N3—C6	1.400 (6)	N41—C41	1.116 (7)
N4—C13	1.341 (5)	C31—C32	1.447 (6)
N4—C14	1.378 (6)	C32—H32A	0.9800
N5—C15	1.310 (6)	C32—H32B	0.9800
N5—C16	1.409 (5)	C32—H32C	0.9800
N6—C23	1.311 (6)	C33—C34	1.441 (9)
N6—C24	1.370 (6)	C34—H34A	0.9800
O1—C5	1.289 (5)	C34—H34B	0.9800
O1—H2O	1.29 (6)	C34—H34C	0.9800
O2—C15	1.267 (5)	C35—C36	1.464 (8)
O2—H2O	1.12 (6)	C36—H36A	0.9800
C1—C2	1.401 (6)	C36—H36B	0.9800
C1—C5	1.477 (7)	C36—H36C	0.9800
C2—C15	1.518 (5)	C37—C38	1.455 (7)
C3—C4	1.379 (6)	C38—H38A	0.9800
C3—H3	0.9500	C38—H38B	0.9800
C4—H4	0.9500	C38—H38C	0.9800
C6—C7	1.389 (6)	C39—C40	1.440 (10)
C6—C14	1.438 (5)	C40—H40A	0.9800
C7—C8	1.412 (7)	C40—H40B	0.9800
C7—H7	0.9500	C40—H40C	0.9800
C8—C9	1.370 (6)	C41—C42	1.447 (10)
C8—H8	0.9500	C42—H42A	0.9800
C9—C10	1.411 (6)	C42—H42B	0.9800
C9—H9	0.9500	C42—H42C	0.9800
C10—C14	1.384 (7)	Cl1—O14	1.407 (4)
C10—C11	1.434 (6)	Cl1—O11	1.420 (5)
C11—C12	1.360 (7)	Cl1—O12	1.428 (4)
C11—H11	0.9500	Cl1—O13	1.433 (5)
C12—C13	1.382 (7)	Cl2—O23	1.392 (5)
C12—H12	0.9500	Cl2—O21	1.417 (5)
C13—H13	0.9500	Cl2—O24	1.423 (5)
C16—C17	1.370 (6)	Cl2—O22	1.432 (4)
C16—C24	1.433 (7)	Cl3—O33	1.408 (4)
C17—C18	1.420 (6)	Cl3—O34	1.424 (4)
C17—H17	0.9500	Cl3—O31	1.434 (4)
C18—C19	1.357 (7)	Cl3—O32	1.450 (4)
			
N3—Cu1—N4	83.36 (15)	C16—C17—C18	119.7 (5)
N4—Cu1—N1	163.76 (14)	C16—C17—H17	120.2
N3—Cu1—N11	163.66 (16)	C18—C17—H17	120.2
N4—Cu1—N11	97.64 (15)	C19—C18—C17	121.8 (4)
N3—Cu1—N1	80.64 (15)	C19—C18—H18	119.1
N11—Cu1—N1	96.94 (15)	C17—C18—H18	119.1
N3—Cu1—N12	103.58 (15)	C18—C19—C20	120.3 (4)
N4—Cu1—N12	100.38 (15)	C18—C19—H19	119.8
N11—Cu1—N12	92.32 (16)	C20—C19—H19	119.8
N1—Cu1—N12	86.12 (15)	C24—C20—C19	118.4 (5)
N5—Cu2—N6	83.48 (15)	C24—C20—C21	117.3 (4)
N5—Cu2—N2	80.04 (15)	C19—C20—C21	124.3 (4)
N6—Cu2—N2	163.51 (16)	C22—C21—C20	119.5 (4)
N5—Cu2—N22	140.74 (16)	C22—C21—H21	120.2
N6—Cu2—N22	99.75 (16)	C20—C21—H21	120.2
N2—Cu2—N22	93.02 (15)	C21—C22—C23	119.4 (5)
N5—Cu2—N21	120.72 (16)	C21—C22—H22	120.3
N6—Cu2—N21	97.88 (15)	C23—C22—H22	120.3
N2—Cu2—N21	90.62 (15)	N6—C23—C22	122.7 (4)
N22—Cu2—N21	97.77 (16)	N6—C23—H23	118.7
C4—N1—C1	121.1 (4)	C22—C23—H23	118.7
C4—N1—Cu1	124.8 (3)	N6—C24—C20	121.9 (4)
C1—N1—Cu1	114.0 (3)	N6—C24—C16	117.3 (3)
C3—N2—C2	120.4 (4)	C20—C24—C16	120.8 (4)
C3—N2—Cu2	124.1 (3)	C31—N11—Cu1	177.0 (5)
C2—N2—Cu2	115.5 (3)	C33—N12—Cu1	138.6 (4)
C5—N3—C6	127.3 (4)	C35—N21—Cu2	165.0 (4)
C5—N3—Cu1	118.1 (3)	C37—N22—Cu2	160.1 (4)
C6—N3—Cu1	114.6 (2)	N11—C31—C32	178.5 (6)
C13—N4—C14	118.2 (4)	C31—C32—H32A	109.5
C13—N4—Cu1	128.6 (3)	C31—C32—H32B	109.5
C14—N4—Cu1	113.2 (3)	H32A—C32—H32B	109.5
C15—N5—C16	125.9 (4)	C31—C32—H32C	109.5
C15—N5—Cu2	119.3 (3)	H32A—C32—H32C	109.5
C16—N5—Cu2	114.2 (3)	H32B—C32—H32C	109.5
C23—N6—C24	119.2 (4)	N12—C33—C34	178.3 (6)
C23—N6—Cu2	128.3 (3)	C33—C34—H34A	109.5
C24—N6—Cu2	112.5 (3)	C33—C34—H34B	109.5
C5—O1—H2O	115 (3)	H34A—C34—H34B	109.5
C15—O2—H2O	116 (3)	C33—C34—H34C	109.5
N1—C1—C2	117.6 (4)	H34A—C34—H34C	109.5
N1—C1—C5	112.8 (4)	H34B—C34—H34C	109.5
C2—C1—C5	129.5 (4)	N21—C35—C36	178.7 (5)
N2—C2—C1	120.1 (3)	C35—C36—H36A	109.5
N2—C2—C15	112.8 (4)	C35—C36—H36B	109.5
C1—C2—C15	127.1 (4)	H36A—C36—H36B	109.5
N2—C3—C4	120.1 (4)	C35—C36—H36C	109.5
N2—C3—H3	119.9	H36A—C36—H36C	109.5
C4—C3—H3	119.9	H36B—C36—H36C	109.5
N1—C4—C3	120.6 (4)	N22—C37—C38	179.3 (6)
N1—C4—H4	119.7	C37—C38—H38A	109.5
C3—C4—H4	119.7	C37—C38—H38B	109.5
O1—C5—N3	123.9 (4)	H38A—C38—H38B	109.5
O1—C5—C1	121.7 (4)	C37—C38—H38C	109.5
N3—C5—C1	114.4 (4)	H38A—C38—H38C	109.5
C7—C6—N3	129.3 (4)	H38B—C38—H38C	109.5
C7—C6—C14	117.8 (4)	N31—C39—C40	179.1 (7)
N3—C6—C14	112.8 (4)	C39—C40—H40A	109.5
C6—C7—C8	120.2 (4)	C39—C40—H40B	109.5
C6—C7—H7	119.9	H40A—C40—H40B	109.5
C8—C7—H7	119.9	C39—C40—H40C	109.5
C9—C8—C7	121.4 (4)	H40A—C40—H40C	109.5
C9—C8—H8	119.3	H40B—C40—H40C	109.5
C7—C8—H8	119.3	N41—C41—C42	176.0 (8)
C8—C9—C10	119.8 (5)	C41—C42—H42A	109.5
C8—C9—H9	120.1	C41—C42—H42B	109.5
C10—C9—H9	120.1	H42A—C42—H42B	109.5
C14—C10—C9	119.3 (4)	C41—C42—H42C	109.5
C14—C10—C11	117.5 (4)	H42A—C42—H42C	109.5
C9—C10—C11	123.1 (5)	H42B—C42—H42C	109.5
C12—C11—C10	118.8 (5)	O14—Cl1—O11	110.9 (4)
C12—C11—H11	120.6	O14—Cl1—O12	109.5 (3)
C10—C11—H11	120.6	O11—Cl1—O12	110.8 (3)
C11—C12—C13	120.6 (4)	O14—Cl1—O13	108.4 (3)
C11—C12—H12	119.7	O11—Cl1—O13	107.4 (3)
C13—C12—H12	119.7	O12—Cl1—O13	109.7 (4)
N4—C13—C12	122.2 (4)	O23—Cl2—O21	111.5 (4)
N4—C13—H13	118.9	O23—Cl2—O24	110.5 (3)
C12—C13—H13	118.9	O21—Cl2—O24	104.3 (4)
N4—C14—C10	122.6 (4)	O23—Cl2—O22	112.1 (3)
N4—C14—C6	116.0 (4)	O21—Cl2—O22	108.4 (3)
C10—C14—C6	121.4 (4)	O24—Cl2—O22	109.7 (3)
O2—C15—N5	125.7 (4)	O33—Cl3—O34	110.8 (3)
O2—C15—C2	122.1 (4)	O33—Cl3—O31	110.6 (3)
N5—C15—C2	112.1 (4)	O34—Cl3—O31	108.7 (3)
C17—C16—N5	128.6 (5)	O33—Cl3—O32	108.3 (3)
C17—C16—C24	119.0 (4)	O34—Cl3—O32	109.7 (3)
N5—C16—C24	112.3 (4)	O31—Cl3—O32	108.8 (3)
			
C4—N1—C1—C2	0.0 (6)	C9—C10—C14—N4	−179.7 (4)
Cu1—N1—C1—C2	−176.7 (3)	C11—C10—C14—N4	−1.6 (7)
C4—N1—C1—C5	178.6 (4)	C9—C10—C14—C6	−0.8 (7)
Cu1—N1—C1—C5	1.8 (4)	C11—C10—C14—C6	177.4 (4)
C3—N2—C2—C1	−1.9 (6)	C7—C6—C14—N4	178.9 (4)
Cu2—N2—C2—C1	179.0 (3)	N3—C6—C14—N4	−1.6 (6)
C3—N2—C2—C15	177.7 (4)	C7—C6—C14—C10	−0.1 (6)
Cu2—N2—C2—C15	−1.4 (5)	N3—C6—C14—C10	179.3 (4)
N1—C1—C2—N2	1.7 (6)	C16—N5—C15—O2	−7.2 (7)
C5—C1—C2—N2	−176.6 (4)	Cu2—N5—C15—O2	−178.6 (3)
N1—C1—C2—C15	−177.8 (4)	C16—N5—C15—C2	176.7 (4)
C5—C1—C2—C15	3.9 (7)	Cu2—N5—C15—C2	5.2 (5)
C2—N2—C3—C4	0.3 (7)	N2—C2—C15—O2	−178.6 (4)
Cu2—N2—C3—C4	179.4 (3)	C1—C2—C15—O2	0.9 (7)
C1—N1—C4—C3	−1.6 (7)	N2—C2—C15—N5	−2.3 (5)
Cu1—N1—C4—C3	174.8 (3)	C1—C2—C15—N5	177.2 (4)
N2—C3—C4—N1	1.4 (7)	C15—N5—C16—C17	6.4 (7)
C6—N3—C5—O1	1.5 (7)	Cu2—N5—C16—C17	178.2 (4)
Cu1—N3—C5—O1	179.8 (3)	C15—N5—C16—C24	−175.3 (4)
C6—N3—C5—C1	179.9 (4)	Cu2—N5—C16—C24	−3.5 (5)
Cu1—N3—C5—C1	−1.8 (5)	N5—C16—C17—C18	179.3 (4)
N1—C1—C5—O1	178.3 (4)	C24—C16—C17—C18	1.1 (6)
C2—C1—C5—O1	−3.3 (7)	C16—C17—C18—C19	−1.6 (7)
N1—C1—C5—N3	−0.1 (5)	C17—C18—C19—C20	1.0 (7)
C2—C1—C5—N3	178.3 (4)	C18—C19—C20—C24	0.0 (7)
C5—N3—C6—C7	0.3 (8)	C18—C19—C20—C21	−179.7 (5)
Cu1—N3—C6—C7	−178.1 (4)	C24—C20—C21—C22	1.1 (7)
C5—N3—C6—C14	−179.1 (4)	C19—C20—C21—C22	−179.2 (5)
Cu1—N3—C6—C14	2.5 (5)	C20—C21—C22—C23	−0.6 (7)
N3—C6—C7—C8	−178.6 (4)	C24—N6—C23—C22	−1.5 (7)
C14—C6—C7—C8	0.8 (7)	Cu2—N6—C23—C22	179.9 (3)
C6—C7—C8—C9	−0.6 (7)	C21—C22—C23—N6	0.8 (8)
C7—C8—C9—C10	−0.3 (7)	C23—N6—C24—C20	2.0 (7)
C8—C9—C10—C14	1.0 (7)	Cu2—N6—C24—C20	−179.2 (3)
C8—C9—C10—C11	−177.1 (5)	C23—N6—C24—C16	−179.1 (4)
C14—C10—C11—C12	0.7 (7)	Cu2—N6—C24—C16	−0.2 (5)
C9—C10—C11—C12	178.8 (4)	C19—C20—C24—N6	178.5 (4)
C10—C11—C12—C13	0.0 (7)	C21—C20—C24—N6	−1.8 (7)
C14—N4—C13—C12	−0.8 (7)	C19—C20—C24—C16	−0.4 (7)
Cu1—N4—C13—C12	−177.8 (3)	C21—C20—C24—C16	179.3 (4)
C11—C12—C13—N4	0.0 (7)	C17—C16—C24—N6	−179.1 (4)
C13—N4—C14—C10	1.6 (6)	N5—C16—C24—N6	2.4 (6)
Cu1—N4—C14—C10	179.0 (3)	C17—C16—C24—C20	−0.2 (7)
C13—N4—C14—C6	−177.4 (4)	N5—C16—C24—C20	−178.6 (4)
Cu1—N4—C14—C6	0.0 (5)		

### [µ-(3-{Hydroxy[(quinolin-8-yl)imino]methyl}pyrazin-2-yl)[(quinolin-8-yl)imino]methanolato]bis[diacetonitrilecopper(II)] tris(perchlorate) acetonitrile disolvate (I) Hydrogen-bond geometry (Å, º)

**Table:**

*D*—H···*A*	*D*—H	H···*A*	*D*···*A*	*D*—H···*A*
O2—H2*O*···O1	1.12 (6)	1.29 (6)	2.397 (4)	169 (6)
C7—H7···O1	0.95	2.35	2.920 (6)	118
C17—H17···O2	0.95	2.32	2.897 (5)	119
C17—H17···O32^i^	0.95	2.36	3.175 (7)	144
C22—H22···O12^ii^	0.95	2.50	3.108 (6)	122
C23—H23···O34^ii^	0.95	2.41	3.255 (7)	148
C34—H34*A*···O24	0.98	2.31	3.130 (10)	140
C34—H34*C*···O11^iii^	0.98	2.56	3.272 (9)	130
C36—H36*A*···O21^iv^	0.98	2.46	3.429 (8)	168
C38—H38*B*···N31^iv^	0.98	2.57	3.454 (10)	150
C40—H40*C*···O32^v^	0.98	2.57	3.516 (8)	162

Symmetry codes: (i) *x*+1, *y*−1, *z*; (ii) *x*, *y*−1, *z*; (iii) *x*, *y*, *z*−1; (iv) −*x*, −*y*, −*z*+1; (v) −*x*, −*y*+1, −*z*+1.
