# Supplementary material for: Syntheses and crystal structures of a new pyrazine dicarboxamide ligand, N 2,N 3-bis­(quinolin-8-yl)pyrazine-2,3-dicarboxamide, and of a copper perchlorate binuclear complex
Source: Acta Crystallogr E Crystallogr Commun. 2020 Feb 14;76(Pt 3):332–8. doi: 10.1107/S2056989020001838 (PMC7057382; doi:10.1107/S2056989020001838)

Search Overview

Search:	search3
Date/Time done:	Wed Feb 05 14:34:22 2020
Database(s):	CSD version 5.41 (November 2019)
Restriction Info:	No refcode restrictions applied
Filters:	None
Percentage Completed:	100%
Number of Hits:	28

Single query used. Search found structures that:

match

Query 1

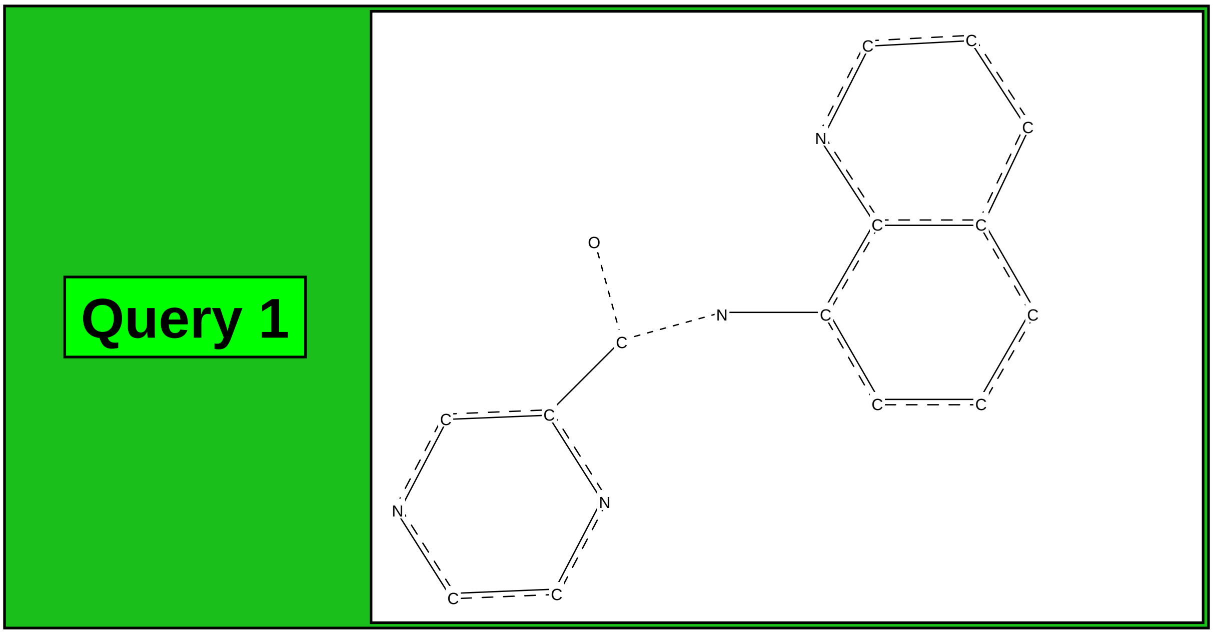

Search: search3 (Wed Feb 05 14:34:22 2020): Hits 1-4

AYIFOF

Reference: M.Soraia Meghdadi, M.Amirnasr, Z.Azarkamanzad, Kurt Schenk Jo, F.Fadaee, A.Amiri, S.Abbasi (2013) *J.Coord.Chem.* , **66,**4330

Formula: $C_{16}\ H_{12}\ Cu_1\ N_4\ O_3,H_2\ O_1$

Compound Name: (acetato)-(N-(quinolin-8-yl)pyrazine-2-carboxamidato)-copper(ii) monohydrate

Space Group:	P-1	***Cell:***	***a*** 6.926(1)	***b*** 9.762(2)	***c*** 12.716(3)
Space Group No.:	2	***(Å,°)***	α 69.78(3)	β 90.07(3)	γ 82.43(3)
R-Factor (%):	4.62	***Temperature(K):*** 292	***Density(g/cm³):*** 1.621		

Parameters

Fragment 1

**ANG1 (A)**	1.032
**DIST1 (D)**	1.234
**DIST2 (D)**	1.335
**DIST4 (D)**	1.390

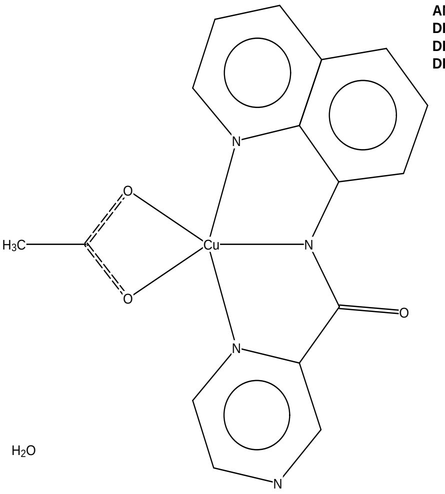

AYIFUL

Reference: M.Soraia Meghdadi, M.Amirnasr, Z.Azarkamanzad, Kurt Schenk Jo, F.Fadaee, A.Amiri, S.Abbasi (2013) *J.Coord.Chem.* , **66,**4330

Formula: $C_{16}\ H_{14}\ N_4\ O_4\ Zn_1$

Compound Name: acetato-aqua-(N-(quinolin-8-yl)pyrazine-2-carboxamidato)-zinc(ii)

Space Group:	P21/c	***Cell:***	***a*** 12.213(0)	***b*** 10.244(0)	***c*** 13.342(0)
Space Group No.:	14	***(Å,°)***	α 90.00	β 105.44(0)	γ 90.00
R-Factor (%):	2.90	***Temperature(K):*** 292	***Density(g/cm³):*** 1.617		

Parameters

Fragment 1

**ANG1 (A)**	9.596
**DIST1 (D)**	1.239
**DIST2 (D)**	1.329
**DIST4 (D)**	1.397

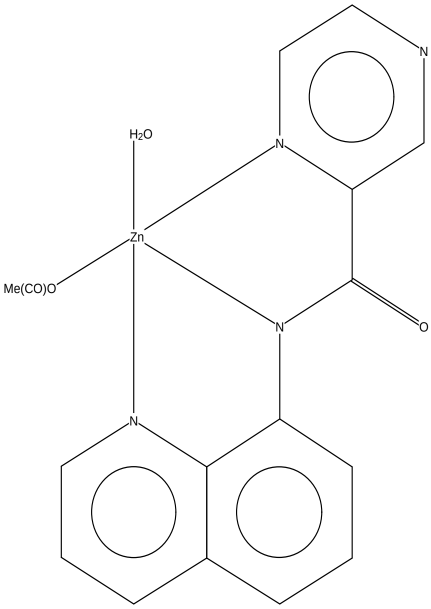

BOFBAC

Reference: H.Stoeckli-Evans, D.S.Cati (2019) *CSD Communication(Private Communication) ,*

Formula: $(C_{14}\ H_9\ Cu_1\ N_7\ O_1)n$

Compound Name: catena-[azido-(μ-N-(quinolin-8-yl)pyrazine-2-carboxamidato)-copper]

Space Group:	Pbca	***Cell:***	***a*** 8.551(0)	***b*** 15.184(1)	***c*** 20.056(1)
Space Group No.:	61	***(Å,°)***	α 90.00	β 90.00	γ 90.00
R-Factor (%):	2.45	***Temperature(K):*** 153	***Density(g/cm³):*** 1.810		

Parameters

Fragment 1

**ANG1 (A)**	6.050
**DIST1 (D)**	1.243
**DIST2 (D)**	1.333
**DIST4 (D)**	1.387

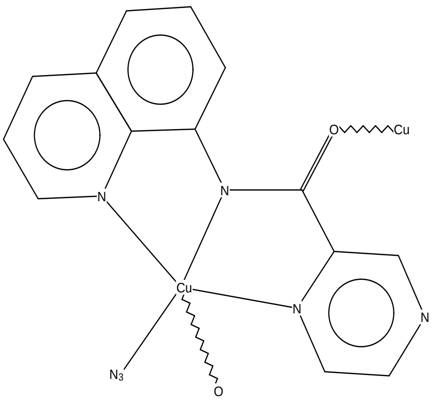

BOFBEG

Reference: H.Stoeckli-Evans, D.S.Cati (2019) *CSD Communication(Private Communication) ,*

Formula: $C_{14}\ H_9\ Cu_1\ N_5\ O_4$

Compound Name: (nitrato)-[N-(quinolin-8-yl)pyrazine-2-carboxamidato]-copper

Space Group:	P21/n	***Cell:***	***a*** 11.091(0)	***b*** 9.148(0)	***c*** 27.335(2)
Space Group No.:	14	***(Å,°)***	α 90.00	β 99.86(0)	γ 90.00
R-Factor (%):	3.05	***Temperature(K):*** 153	***Density(g/cm³):*** 1.822		

Parameters

Fragment 1

**ANG1 (A)**	12.666
**DIST1 (D)**	1.244
**DIST2 (D)**	1.340
**DIST4 (D)**	1.384

Fragment 2

**ANG1 (A)**	5.405
**DIST1 (D)**	1.233
**DIST2 (D)**	1.341
**DIST4 (D)**	1.390

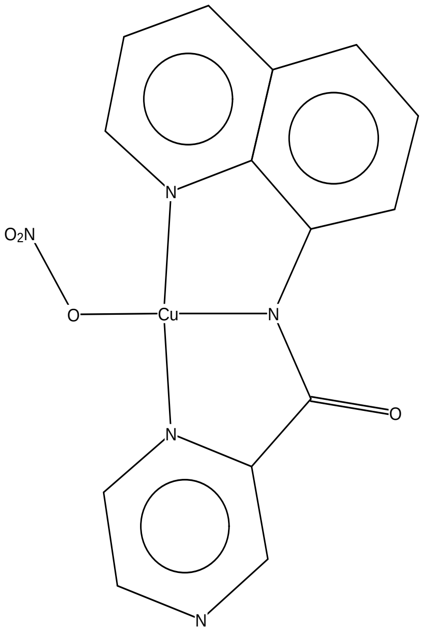

Search: search3 (Wed Feb 05 14:34:22 2020): Hits 5-8

BOLJOD

Reference: Hongbo Zhou, Yingying Wang, Fangyou Mou, Xiaoping Shen, Yashu Liu (2014) *New J.Chem.* ,**38,**5925

Formula: $C_{37}\ H_{33}\ Fe_1\ Mn_1\ N_9\ O_6$

Compound Name: (μ_2-Cyano)-aqua-bis(cyano)-(((5-methylpyrazin-2-yl)carbonyl)(quinolin-8-yl)amino)-(2,2'-(propane-1,2-diylbis((nitrilo)methylylidene))bis(6-methoxyphenolato))-manganese(iii)-iron(iii)

Space Group:	P21/n	***Cell:***	***a*** 11.985(3)	***b*** 14.249(4)	***c*** 22.419(6)
Space Group No.:	14	***(Å,°)***	α 90.00	β 95.53(0)	γ 90.00

R-Factor (%): 5.16 ***Temperature(K):*** 173 ***Density(g/cm³):*** 1.413

Parameters

Fragment 1

**ANG1 (A)**	14.112
**DIST1 (D)**	1.218
**DIST2 (D)**	1.361
**DIST4 (D)**	1.396

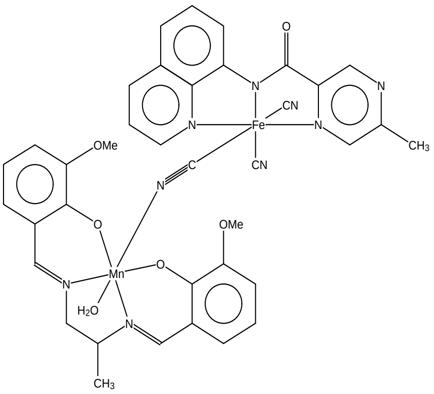

BOLJUJ

Reference: Hongbo Zhou, Yingying Wang, Fangyou Mou, Xiaoping Shen, Yashu Liu (2014) *New J.Chem.* ,**38,**5925

Formula: $C_{36}\ H_{31}\ Fe_1\ Mn_1\ N_9\ O_6$

Compound Name: (μ_2-Cyano)-aqua-bis(cyano)-(((5-methylpyrazin-2-yl)carbonyl)(quinolin-8-yl)amino)-(2,2'-(ethane-1,2-diylbis((nitrilo)methylylidene))bis(6-methoxyphenolato))-manganese(iii)-iron(iii)

Space Group:	P-1	***Cell:***	***a*** 11.415(5)	***b*** 13.273(5)	***c*** 14.081(6)
Space Group No.:	2	***(Å,°)***	α 65.73(0)	β 67.53(0)	γ 71.71(0)

R-Factor (%): 6.78 ***Temperature(K):*** 173 ***Density(g/cm³):*** 1.498

Parameters

Fragment 1

**ANG1 (A)**	9.813
**DIST1 (D)**	1.224
**DIST2 (D)**	1.371
**DIST4 (D)**	1.400

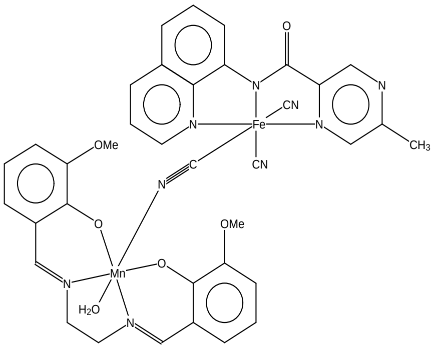

BOLKIY

Reference: Hongbo Zhou, Yingying Wang, Fangyou Mou, Xiaoping Shen, Yashu Liu (2014) *New J.Chem.* ,**38,**5925

Formula: $C_{36}\ H_{31}\ Fe_1\ Mn_1\ N_9\ O_6,0.72(C_2\ H_3\ N_1),H_2\ O_1$

Compound Name: (μ_2-Cyano)-aqua-bis(cyano)-(((5-methylpyrazin-2-yl)carbonyl)(quinolin-8-yl)amino)-(2,2'-(ethane-1,2-diylbis((nitrilo)methylylidene))bis(6-methoxyphenolato))-manganese(iii)-iron(iii) acetonitrile solvate monohydrate

Space Group:	P21/n	***Cell:***	***a*** 11.933(3)	***b*** 14.282(3)	***c*** 22.324(4)
Space Group No.:	14	***(Å,°)***	α 90.00	β 94.29(5)	γ 90.00

R-Factor (%): 6.84 ***Temperature(K):*** 173 ***Density(g/cm³):*** 1.478

Parameters

Fragment 1

**ANG1 (A)**	15.091
**DIST1 (D)**	1.240
**DIST2 (D)**	1.356
**DIST4 (D)**	1.404

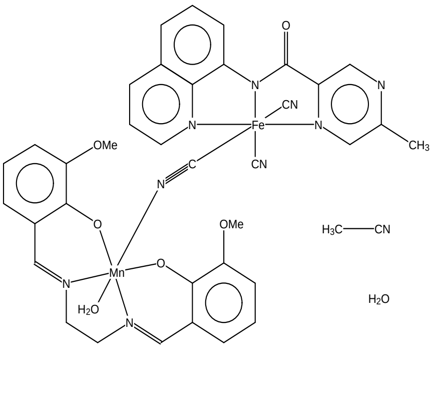

DIBMIM

Reference: A.Panja (2013) *J.Coord.Chem.* ,**66,**2178

Formula: $C_{36}\ H_{30}\ Co_1\ Fe_2\ N_{14}\ O_6,2(H_2\ O_1)$

Compound Name: bis(μ_2-Cyano)-diaqua-tetracyano-bis(N-(quinolin-8-yl)pyrazine-2-carboxamide)-bis(methanol)-cobalt-di-iron dihydrate

Space Group:	C2/c	***Cell:***	***a*** 20.232(0)	***b*** 14.393(0)	***c*** 15.067(0)
Space Group No.:	15	***(Å,°)***	α 90.00	β 113.15(0)	γ 90.00

R-Factor (%): 4.16 ***Temperature(K):*** 150 ***Density(g/cm³):*** 1.583

Parameters

Fragment 1

**ANG1 (A)**	2.133
**DIST1 (D)**	1.230
**DIST2 (D)**	1.359
**DIST4 (D)**	1.395

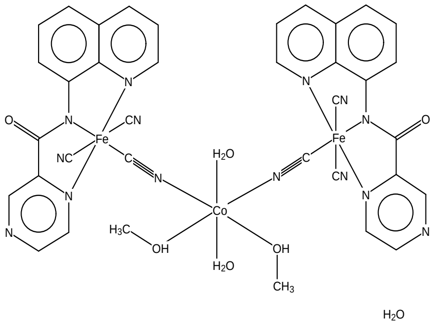

Search: search3 (Wed Feb 05 14:34:22 2020): Hits 9-12

EFODIP

Reference:	D.S.Cati, H.Stoeckli-Evans (2019) *Acta Crystallogr.,Sect.E:Cryst.Commun.* ,**75,**755
Formula:	$C_{14}\ H_{10}\ N_4\ O_1$
Compound Name:	N-(quinolin-8-yl)pyrazine-2-carboxamide

Space Group:	Cc	***Cell:***	***a*** 11.505(0)	***b*** 23.410(3)	***c*** 13.412(1)	
Space Group No.:	9	***(Å,°)***	α 90.00	β 104.31(0)	γ 90.00	
R-Factor (%):	5.94	***Temperature(K):*** 223	***Density(g/cm³):*** 1.425			

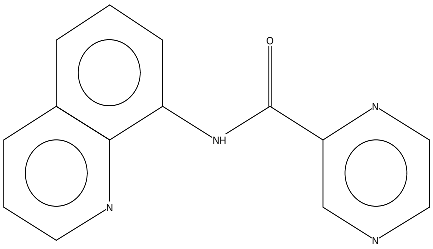

Parameters

Fragment 1	
**ANG1 (A)**	3.091
**DIST1 (D)**	1.224
**DIST2 (D)**	1.366
**DIST4 (D)**	1.420
Fragment 2	
**ANG1 (A)**	4.133
**DIST1 (D)**	1.224
**DIST2 (D)**	1.346
**DIST4 (D)**	1.400
Fragment 3	
**ANG1 (A)**	4.528
**DIST1 (D)**	1.230
**DIST2 (D)**	1.351
**DIST4 (D)**	1.397

EFODOV

Reference:	D.S.Cati, H.Stoeckli-Evans (2019) *Acta Crystallogr.,Sect.E:Cryst.Commun.* ,**75,**755
Formula:	$C_{42}\ H_{44}\ Cu_4\ N_8\ O_{16}$,2($C_1\ H_4\ O_1$)
Compound Name:	hexakis(μ-acetato)-bis(methanol)-bis(N-(quniolin-8-yl)pyrazine-2-carboxamide)-tetra-copper(ii) methanol solvate

Space Group:	P-1	***Cell:***	***a*** 8.149(0)	***b*** 11.213(0)	***c*** 14.266(1)
Space Group No.:	2	***(Å,°)***	α 98.35(0)	β 93.67(1)	γ 103.58(0)
R-Factor (%):	3.10	***Temperature(K):*** 153	***Density(g/cm³):*** 1.645		

Parameters

Fragment 1	
**ANG1 (A)**	7.463
**DIST1 (D)**	1.249
**DIST2 (D)**	1.332
**DIST4 (D)**	1.388

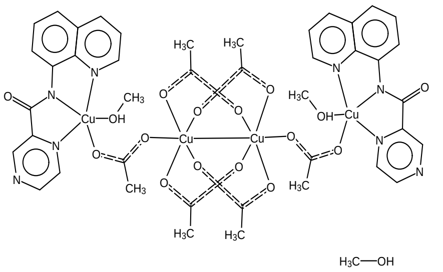

JIVGEB

Reference:	Jae Il Kim, Houng Sik Yoo, Eui Kwan Koh, Chang Seop Hong (2007) *Inorg.Chem.* ,**46,**10461
Formula:	$C_{18}\ H_{11}\ Fe_1\ N_7\ O_1\ ^{1-}$,$C_{24}\ H_{20}\ P_1\ ^{1+}$,2.5($C_1\ H_4\ O_1$),0.5($H_2\ O_1$)
Compound Name:	Tetraphenylphosphonium mer-tricyano-(8-((5-methylpyrazin-2-yl) carbonylamido)quinoline-N,N',N'')-iron(iii) methanol solvate hemihydrate

Space Group:	C2/c	***Cell:***	***a*** 37.651(1)	***b*** 9.284(0)	***c*** 27.944(0)
Space Group No.:	15	***(Å,°)***	α 90.00	β 122.20(0)	γ 90.00
R-Factor (%):	6.56	***Temperature(K):*** 130	***Density(g/cm³):*** 1.327		

Parameters

Fragment 1	
**ANG1 (A)**	4.391
**DIST1 (D)**	1.235
**DIST2 (D)**	1.352
**DIST4 (D)**	1.394

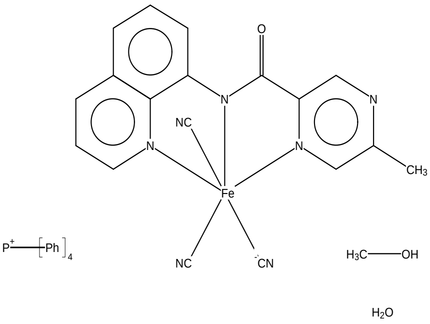

JIVGIF

Reference:	Jae Il Kim, Houng Sik Yoo, Eui Kwan Koh, Chang Seop Hong (2007) *Inorg.Chem.* ,**46,**10461
Formula:	($C_{38}\ H_{31}\ Fe_1\ Mn_1\ N_9\ O_3$)n,n($C_1\ H_4\ O_1$),n($C_2\ H_3\ N_1$)
Compound Name:	catena-(bis(μ_2-Cyano)-cyano-(8-((5-methylpyrazin-2-yl)carbonylamido) quinoline-N,N',N'')-(N,N'-(trans-cyclohexane-1,2-diyl)-bis(salicylideneaminato)-N,N',O,O')-iron(iii)-manganese(iii) acetonitrile methanol solvate)

Space Group:	P21/c	***Cell:***	***a*** 12.486(0)	***b*** 20.344(0)	***c*** 14.947(0)
Space Group No.:	14	***(Å,°)***	α 90.00	β 92.22(0)	γ 90.00
R-Factor (%):	7.85	***Temperature(K):*** 130	***Density(g/cm³):*** 1.480		

Parameters

Fragment 1	
**ANG1 (A)**	9.465
**DIST1 (D)**	1.234
**DIST2 (D)**	1.353
**DIST4 (D)**	1.395

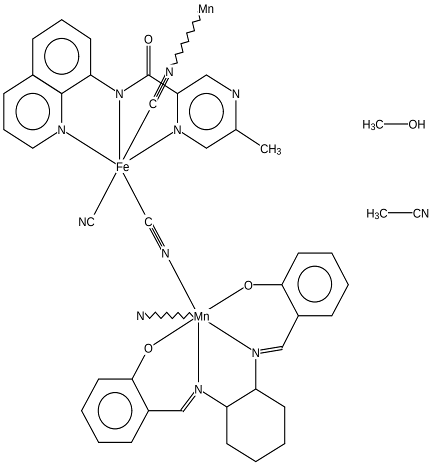

Search: search3 (Wed Feb 05 14:34:22 2020): Hits 13-16

JIVGOL

Reference: Jae Il Kim, Houng Sik Yoo, Eui Kwan Koh, Chang Seop Hong (2007) *Inorg.Chem.* ,**46,**10461

Formula: C_{34} H_{27} Fe_1 Mn_1 N_9 O_4,H_2 O_1

Compound Name: (μ_2-Cyano)-aqua-dicyano-(8-((5-methylpyrazin-2-yl)carbonylamido) quinoline-N,N',N'')-(N,N'-(ethylene)-bis(salicylideneaminato)-N,N',O,O')-iron(iii)-manganese(iii) monohydrate

Space Group:	Cc	***Cell:***	***a*** 10.766(1)	***b*** 23.771(2)	***c*** 13.832(1)
Space Group No.:	9	***(Å,°)***	α 90.00	β 110.58(0)	γ 90.00

R-Factor (%): 3.31 ***Temperature(K):*** 293 ***Density(g/cm^3):*** 1.512

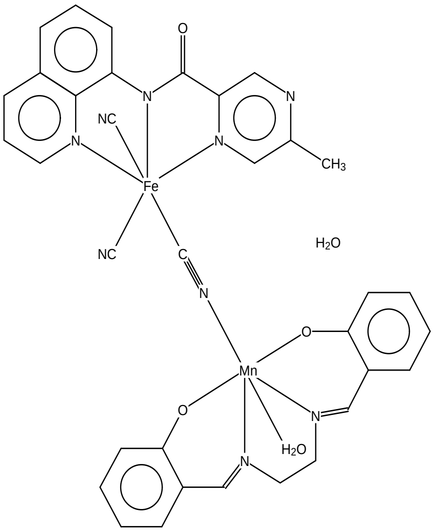

Parameters

Fragment 1

**ANG1 (A)**	0.413
**DIST1 (D)**	1.223
**DIST2 (D)**	1.356
**DIST4 (D)**	1.391

MUPWEB

Reference: Anangamohan Panja (2014) *J.Indian Chem.Soc.* ,**91,**237

Formula: 2(C_{24} H_{20} P_1 $^{1+}$),C_{17} H_9 Fe_1 N_7 O_1 $^{2-}$,6(H_2 O_1)

Compound Name: bis(Tetraphenylphosphonium) tricyano-(N-(quinolin-8-yl)pyrazine-2-carboxamidato)-iron hexahydrate

Space Group:	P21/c	***Cell:***	***a*** 16.682(0)	***b*** 9.162(0)	***c*** 39.046(1)
Space Group No.:	14	***(Å,°)***	α 90.00	β 95.25(0)	γ 90.00

R-Factor (%): 7.75 ***Temperature(K):*** 150 ***Density(g/cm^3):*** 1.308

Parameters

Fragment 1

**ANG1 (A)**	5.816
**DIST1 (D)**	1.264
**DIST2 (D)**	1.328
**DIST4 (D)**	1.402

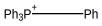

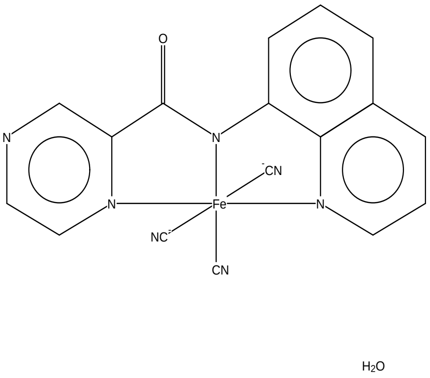

MUPWIF

Reference: Anangamohan Panja (2014) *J.Indian Chem.Soc.* ,**91,**237

Formula: C_{16} H_{36} N_1 $^{1+}$,C_{17} H_9 Fe_1 N_7 O_1 $^{1-}$,1.5(H_2 O_1)

Compound Name: Tetra-n-butylammonium tricyano-(N-(quinolin-8-yl)pyrazine-2-carboxamidato)-iron(iii) sesquihydrate

Space Group:	P21/n	***Cell:***	***a*** 13.690(4)	***b*** 14.896(4)	***c*** 17.373(5)
Space Group No.:	14	***(Å,°)***	α 90.00	β 100.70(0)	γ 90.00

R-Factor (%): 6.46 ***Temperature(K):*** 150 ***Density(g/cm^3):*** 1.245

Parameters

Fragment 1

**ANG1 (A)**	11.349
**DIST1 (D)**	1.240
**DIST2 (D)**	1.364
**DIST4 (D)**	1.392

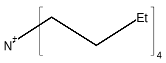

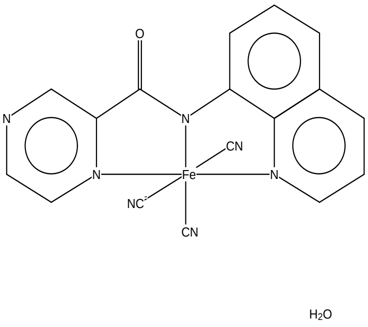

MUPXIG

Reference: Anangamohan Panja (2014) *J.Indian Chem.Soc.* ,**91,**619

Formula: C_{26} H_{16} Co_1 N_6,C_{17} H_9 Fe_1 N_7 O_1,2.5(H_2 O_1)

Compound Name: Dicyano-bis(1,10-phenanthroline)-cobalt tricyano-(N-(quinolin-8-yl) pyrazine-2-carboxamidato)-iron hydrate

Space Group:	P-1	***Cell:***	***a*** 10.987(5)	***b*** 12.540(5)	***c*** 16.424(5)
Space Group No.:	2	***(Å,°)***	α 67.54(0)	β 75.54(0)	γ 88.01(0)

R-Factor (%): 7.58 ***Temperature(K):*** 293 ***Density(g/cm^3):*** 1.479

Parameters

Fragment 1

**ANG1 (A)**	8.586
**DIST1 (D)**	1.231
**DIST2 (D)**	1.354
**DIST4 (D)**	1.396

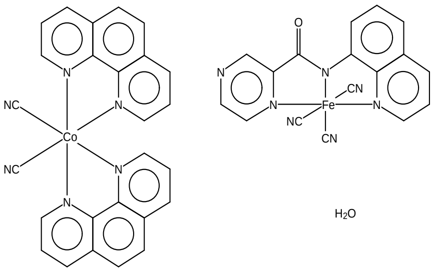

Search: search3 (Wed Feb 05 14:34:22 2020): Hits 17-20

NIVDII

Reference: H.Stoeckli-Evans (2019)
CSD Communication(Private Communication) ,

Formula: $C_{33} H_{26} N_{10} O_4, C_2 H_3 N_1$

Compound Name: $N^3,N^{3'}$-(propane-1,3-diyl)bis(N^2-(quinolin-8-yl)pyrazine-2,3-dicarboxamide) acetonitrile solvate

Space Group:	P21/c	***Cell:***	***a*** 9.151(1)	***b*** 18.313(2)	***c*** 18.957(3)
Space Group No.:	14	***(Å,°)***	α 90.00	β 97.19(2)	γ 90.00

R-Factor (%): 4.94 ***Temperature(K):*** 153 ***Density(g/cm³):*** 1.407

Parameters

Fragment 1
ANG1 (A) 11.225
DIST1 (D) 1.236
DIST2 (D) 1.354
DIST4 (D) 1.409

Fragment 2
ANG1 (A) 20.002
DIST1 (D) 1.249
DIST2 (D) 1.342
DIST4 (D) 1.401

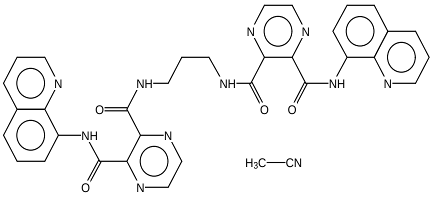

NIVFUW

Reference: D.S.Cati, H.Stoeckli-Evans (2019)
CSD Communication(Private Communication) ,

Formula: $C_{17} H_{14} N_4 O_3$

Compound Name: ethyl 3-((quinolin-8-yl)carbamoyl)pyrazine-2-carboxylate

Space Group:	P21/n	***Cell:***	***a*** 20.844(15)	***b*** 7.636(6)	***c*** 21.110(14)
Space Group No.:	14	***(Å,°)***	α 90.00	β 114.32(7)	γ 90.00

R-Factor (%): 5.94 ***Temperature(K):*** 223 ***Density(g/cm³):*** 1.398

Parameters

Fragment 1
ANG1 (A) 1.248
DIST1 (D) 1.224
DIST2 (D) 1.343
DIST4 (D) 1.398

Fragment 2
ANG1 (A) 5.580
DIST1 (D) 1.220
DIST2 (D) 1.345
DIST4 (D) 1.405

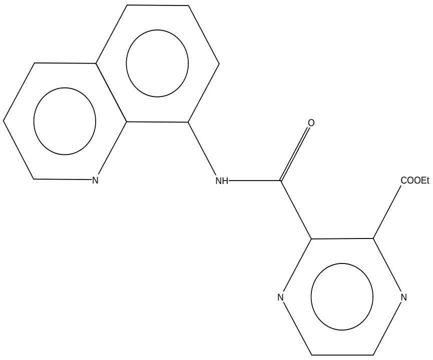

NIVGAD

Reference: D.S.Cati, H.Stoeckli-Evans (2019)
CSD Communication(Private Communication) ,

Formula: $C_{32} H_{22} Cu_2 N_{10} O_{12}$

Compound Name: bis(μ-methyl 3-((quinolin-8-yl)carbamoyl)pyrazine-2-carboxylatato)-dinitrato-di-copper

Space Group:	P-1	***Cell:***	***a*** 8.988(1)	***b*** 10.213(1)	***c*** 11.013(1)
Space Group No.:	2	***(Å,°)***	α 113.27(1)	β 97.90(1)	γ 108.19(1)

R-Factor (%): 5.67 ***Temperature(K):*** 153 ***Density(g/cm³):*** 1.707

Parameters

Fragment 1
ANG1 (A) 1.682
DIST1 (D) 1.237
DIST2 (D) 1.342
DIST4 (D) 1.388

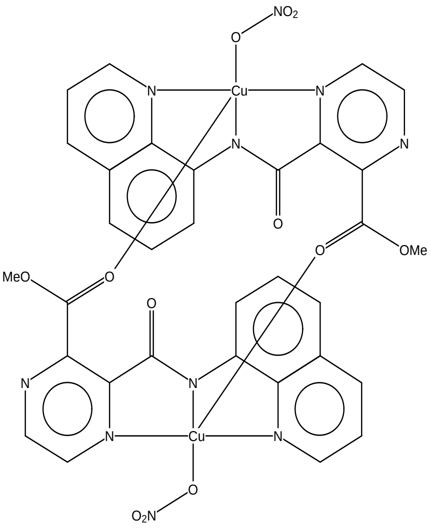

NOQWOH

Reference: Hongbo Zhou, Yingying Wang, Fangyou Mou, Xiaoping Shen, Yashu Liu (2015) *Polyhedron* ,**85,**457

Formula: $C_{18} H_{11} Fe_1 N_7 O_1{}^{1-}, C_{24} H_{20} P_1{}^{1+}, 4(H_2 O_1)$

Compound Name: Tetraphenylphosphonium tricyano-(5-methyl-N-(quinolin-8-yl)pyrazine-2-carboxamidato)-iron(iii) tetrahydrate

Space Group:	P21/c	***Cell:***	***a*** 15.654(3)	***b*** 13.622(3)	***c*** 18.605(4)
Space Group No.:	14	***(Å,°)***	α 90.00	β 104.18(3)	γ 90.00

R-Factor (%): 7.61 ***Temperature(K):*** 173 ***Density(g/cm³):*** 1.396

Parameters

Fragment 1
ANG1 (A) 8.276
DIST1 (D) 1.220
DIST2 (D) 1.360
DIST4 (D) 1.430

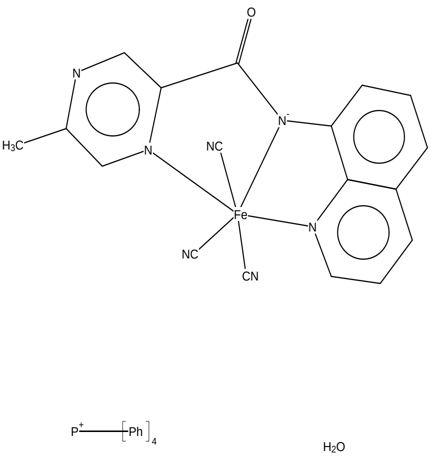

NOQWUN

Reference: Hongbo Zhou, Yingying Wang, Fangyou Mou, Xiaoping Shen, Yashu Liu (2015) *Polyhedron* ,**85**,457

Formula: $C_{36} H_{40} Mn_2 N_4 O_{10},2(C_{18} H_{11} Fe_1 N_7 O_1),2(H_2 O_1)$

Compound Name: bis(μ_2-2,2'-(Ethane-1,2-diylbis((nitrilo)methylylidene))bis(4-methoxyphenolato))-diaqua-di-manganese bis(tricyano-(5-methyl-N-(quinolin-8-yl)pyrazine-2-carboxamidato)-iron) dihydrate

Space Group:	C2/c	***Cell:***	***a*** 29.730(2)	***b*** 17.168(3)	***c*** 14.363(2)		
Space Group No.:	15	***(Å,°)***	α 90.00	β 108.49(3)	γ 90.00		
R-Factor (%):	3.45	***Temperature(K):***	173	***Density(g/cm³):***	1.556		

Parameters

Fragment 1

**ANG1 (A)**	9.157
**DIST1 (D)**	1.230
**DIST2 (D)**	1.356
**DIST4 (D)**	1.394

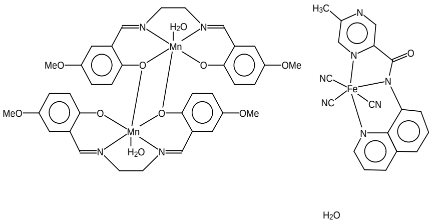

NOQXAU

Reference: Hongbo Zhou, Yingying Wang, Fangyou Mou, Xiaoping Shen, Yashu Liu (2015) *Polyhedron* ,**85**,457

Formula: $(C_{36} H_{29} Fe_1 Mn_1 N_9 O_3)n,n(C_2 H_3 N_1),n(H_2 O_1)$

Compound Name: catena-(bis(μ_2-Cyano-C,N)-cyano-(2,2'-(ethane-1,2-diylbis(nitrilomethylylidene))bis(4-methylphenolato))-(5-methyl-N-(quinolin-8-yl)pyrazine-2-carboxamide)-iron-manganese acetonitrile solvate monohydrate)

Space Group:	P212121	***Cell:***	***a*** 12.175(3)	***b*** 13.545(2)	***c*** 22.344(3)
Space Group No.:	19	***(Å,°)***	α 90.00	β 90.00	γ 90.00
R-Factor (%):	2.48	***Temperature(K):***	173	***Density(g/cm³):***	1.452

Parameters

Fragment 1

**ANG1 (A)**	10.689
**DIST1 (D)**	1.237
**DIST2 (D)**	1.356
**DIST4 (D)**	1.393

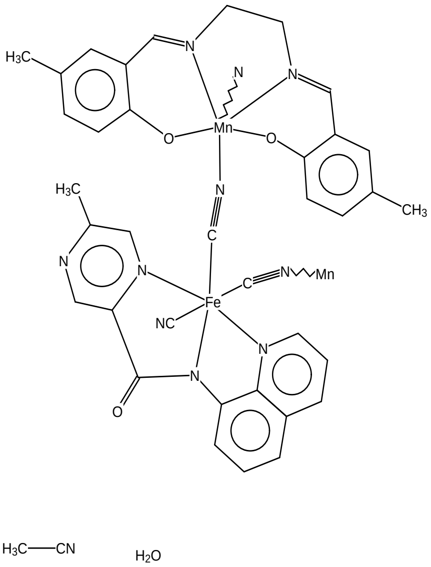

SODZOB

Reference: Jae Il Kim, Jung Hee Yoon, Hyun Young Kwak, Eui Kwan Koh, Chang Seop Hong (2008) *Eur.J.Inorg.Chem.* ,2756

Formula: $C_{41} H_{25} Cl_1 Fe_1 Mn_1 N_{11} O_1,C_1 H_4 O_1$

Compound Name: (μ_2-Cyano)-chloro-dicyano-bis(1,10-phenanthroline)-(8-(pyrazine-2-carboxamido)quinolinato)-iron(iii)-manganese(ii) methanol solvate

Space Group:	P-1	***Cell:***	***a*** 11.490(2)	***b*** 12.608(2)	***c*** 15.432(3)
Space Group No.:	2	***(Å,°)***	α 72.97(0)	β 69.48(0)	γ 67.22(0)
R-Factor (%):	6.87	***Temperature(K):***	293	***Density(g/cm³):***	1.515

Parameters

Fragment 1

**ANG1 (A)**	10.378
**DIST1 (D)**	1.235
**DIST2 (D)**	1.351
**DIST4 (D)**	1.397

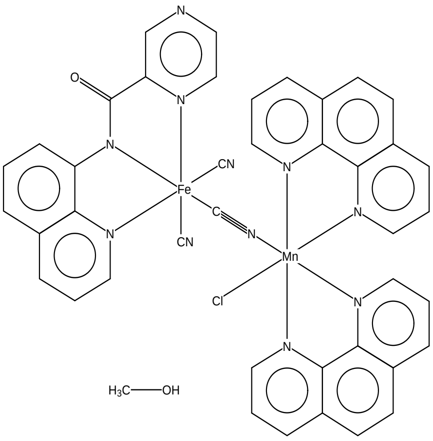

SODZUH

Reference: Jae Il Kim, Jung Hee Yoon, Hyun Young Kwak, Eui Kwan Koh, Chang Seop Hong (2008) *Eur.J.Inorg.Chem.* ,2756

Formula: $C_{41} H_{25} Br_1 Fe_1 Mn_1 N_{11} O_1,C_1 H_4 O_1$

Compound Name: (μ_2-Cyano)-bromo-dicyano-bis(1,10-phenanthroline)-(8-(pyrazine-2-carboxamido)quinolinato)-iron(iii)-manganese(ii) methanol solvate

Space Group:	P-1	***Cell:***	***a*** 11.284(0)	***b*** 12.804(0)	***c*** 15.446(0)
Space Group No.:	2	***(Å,°)***	α 73.10(0)	β 70.40(0)	γ 66.22(0)
R-Factor (%):	5.57	***Temperature(K):***	293	***Density(g/cm³):***	1.598

Parameters

Fragment 1

**ANG1 (A)**	7.498
**DIST1 (D)**	1.228
**DIST2 (D)**	1.360
**DIST4 (D)**	1.399

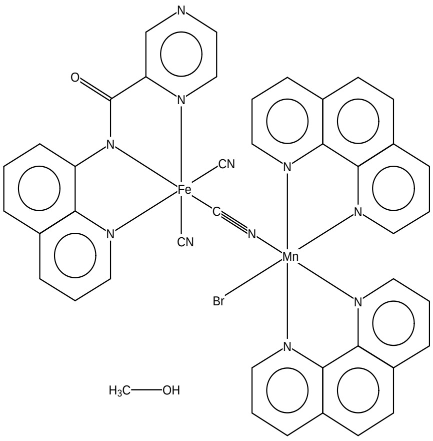

Search: search3 (Wed Feb 05 14:34:22 2020): Hits 25-28

SOFBAR

Reference: Jae Il Kim, Jung Hee Yoon, Hyun Young Kwak, Eui Kwan Koh, Chang Seop Hong (2008) *Eur.J.Inorg.Chem.* ,2756

Formula: $C_{42}\,H_{27}\,Cl_1\,Fe_1\,Mn_1\,N_{11}\,O_1,C_1\,H_4\,O_1$

Compound Name: (μ_2-Cyano)-chloro-dicyano-bis(1,10-phenanthroline)-(8-(5-methylpyrazine-2-carboxamido)quinolinato)-iron(iii)-manganese(ii) methanol solvate

Space Group:	P-1	***Cell:***	***a*** 11.909(0)	***b*** 12.081(0)	***c*** 16.070(0)
Space Group No.:	2	***(Å,°)***	α 74.45(0)	β 70.77(0)	γ 67.35(0)

R-Factor (%): 5.06 ***Temperature(K):*** 293 ***Density(g/cm^3):*** 1.470

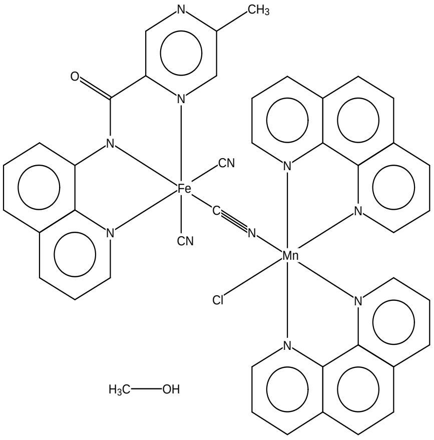

Parameters

Fragment 1

**ANG1 (A)**	8.000
**DIST1 (D)**	1.227
**DIST2 (D)**	1.356
**DIST4 (D)**	1.400

VIRJUC

Reference: Jae Il Kim, Houng Sik Yoo, Eui Kwan Koh, Hyoung Chan Kim, Chang Seop Hong (2007) *Inorg.Chem.* ,**46,**8481

Formula: $C_{24}\,H_{20}\,P_1\,^{1+},C_{17}\,H_9\,Fe_1\,N_7\,O_1\,^{1-}$

Compound Name: Tetraphenylphosphonium tricyano-(8-(pyrazine-2-carboxamido) quinolinate-N,N',N'')-iron(iii)

Space Group:	P21/c	***Cell:***	***a*** 8.602(0)	***b*** 15.053(0)	***c*** 27.764(0)
Space Group No.:	14	***(Å,°)***	α 90.00	β 108.05(0)	γ 90.00

R-Factor (%): 4.17 ***Temperature(K):*** 130 ***Density(g/cm^3):*** 1.404

Parameters

Fragment 1

**ANG1 (A)**	7.618
**DIST1 (D)**	1.234
**DIST2 (D)**	1.358
**DIST4 (D)**	1.401

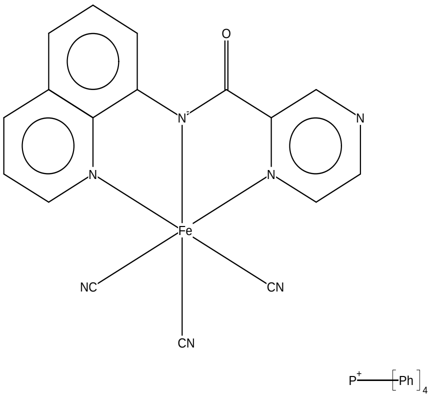

VIRKAJ

Reference: Jae Il Kim, Houng Sik Yoo, Eui Kwan Koh, Hyoung Chan Kim, Chang Seop Hong (2007) *Inorg.Chem.* ,**46,**8481

Formula: $(C_{33}\,H_{23}\,Fe_1\,Mn_1\,N_9\,O_3)n,4n(H_2\,O_1)$

Compound Name: catena-(bis(μ_2-Cyano-C,N)-cyano-(8-(pyrazine-2-carboxamido) quinolinate-N,N',N'')-(N,N'-ethylenebis(salicylideneiminato)-N,N',O,O')-iron(iii)-manganese(iii) tetrahydrate)

Space Group:	P21/n	***Cell:***	***a*** 14.511(0)	***b*** 12.197(0)	***c*** 19.341(0)
Space Group No.:	14	***(Å,°)***	α 90.00	β 91.45(0)	γ 90.00

R-Factor (%): 6.48 ***Temperature(K):*** 130 ***Density(g/cm^3):*** 1.507

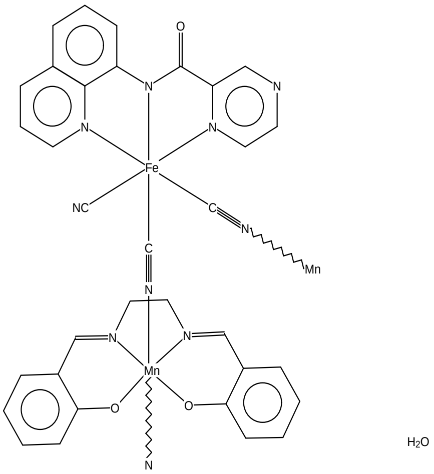

Parameters

Fragment 1

**ANG1 (A)**	5.257
**DIST1 (D)**	1.221
**DIST2 (D)**	1.357
**DIST4 (D)**	1.392

XABHOA

Reference: Daopeng Zhang, Ping Wang, Kexun Chen, Xia Chen (2014) *J.Chem.Sci.(Bangalore,India)* ,**126,**1665

Formula: $C_{37}\,H_{31}\,Fe_1\,Mn_1\,N_9\,O_3,C_1\,H_4\,O_1$

Compound Name: bis(cyano)-(2,2'-((2,3-dimethylbutane-2,3-diyl)bis((nitrilo)methylylidene)) diphenolatato)-(μ-cyano)-(((pyrazin-2-yl)carbonyl)(quinolin-8-yl)azanide)-manganese(iii)-iron(iii) methanol solvate

Space Group:	C2/c	***Cell:***	***a*** 24.939(8)	***b*** 12.805(3)	***c*** 22.346(6)
Space Group No.:	15	***(Å,°)***	α 90.00	β 92.61(0)	γ 90.00

R-Factor (%): 5.32 ***Temperature(K):*** 293 ***Density(g/cm^3):*** 1.477

Parameters

Fragment 1

**ANG1 (A)**	8.229
**DIST1 (D)**	1.231
**DIST2 (D)**	1.368
**DIST4 (D)**	1.385

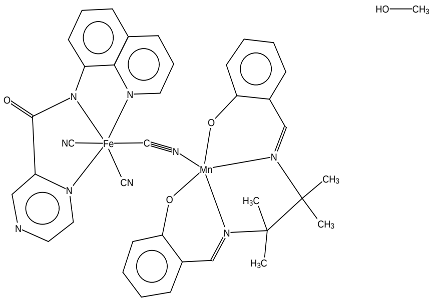

Supplement: Supplementary file 4 [file e-76-00332-sup4.pdf]
